# Novel Roles of Formin mDia2 in Lamellipodia and Filopodia Formation in Motile Cells 

**DOI:** 10.1371/journal.pbio.0050317

**Published:** 2007-11-27

**Authors:** Changsong Yang, Lubov Czech, Silke Gerboth, Shin-ichiro Kojima, Giorgio Scita, Tatyana Svitkina

**Affiliations:** 1 Department of Biology, University of Pennsylvania, Philadelphia, Pennsylvania, United States of America; 2 Department of Cell and Molecular Biology, Feinberg School of Medicine, Northwestern University, Chicago, Illinois, United States of America; 3 The Italian Foundation for Cancer Research (FIRC) Institute for Molecular Oncology, Milan, Italy; 4 Department of Experimental Oncology, European Institute of Oncology, Milan, Italy; Dana-Farber Cancer Institute, United States of America

## Abstract

Actin polymerization-driven protrusion of the leading edge is a key element of cell motility. The important actin nucleators formins and the Arp2/3 complex are believed to have nonoverlapping functions in inducing actin filament bundles in filopodia and dendritic networks in lamellipodia, respectively. We tested this idea by investigating the role of mDia2 formin in leading-edge protrusion by loss-of-function and gain-of-function approaches. Unexpectedly, mDia2 depletion by short interfering RNA (siRNA) severely inhibited lamellipodia. Structural analysis of the actin network in the few remaining lamellipodia suggested an mDia2 role in generation of long filaments. Consistently, constitutively active mDia2 (ΔGBD-mDia2) induced accumulation of long actin filaments in lamellipodia and increased persistence of lamellipodial protrusion. Depletion of mDia2 also inhibited filopodia, whereas expression of ΔGBD-mDia2 promoted their formation. Correlative light and electron microscopy showed that ΔGBD-mDia2–induced filopodia were formed from lamellipodial network through gradual convergence of long lamellipodial filaments into bundles. Efficient filopodia induction required mDia2 targeting to the membrane, likely through a scaffolding protein Abi1. Furthermore, mDia2 and Abi1 interacted through the N-terminal regulatory sequences of mDia2 and the SH3-containing Abi1 sequences. We propose that mDia2 plays an important role in formation of lamellipodia by nucleating and/or protecting from capping lamellipodial actin filaments, which subsequently exhibit high tendency to converge into filopodia.

## Author Summary

Cell motility is a cyclic process, with the protrusion of the leading edge followed by retraction of the rear. Protrusion is driven by polymerization of actin filaments, with the spatial organization of these filaments determining the shape of the protrusions. For example, the spike-like filopodia contain bundles of long actin filaments, whereas the sheet-like lamellipodia contain branched actin networks. In biochemical assays, two stimulators of actin polymerization, Arp2/3 complex and formins, induce branched or individual filaments, respectively. In cells, Arp2/3 complex and formins also appear to be implicated in the formation of lamellipodia and filopodia, respectively. However, when we investigated the role of mDia2 formin by functional approaches, we unexpectedly found that it is essential, not only for filopodia, but also for lamellipodia. Moreover, functions of mDia2 in lamellipodia and filopodia appeared intimately linked. We recorded behavior of cells by light microscopy and then used electron microscopy to study actin architecture in the same cells. We found that an activated form of mDia2 was first recruited to lamellipodia, where it induced many long, unbranched filaments, and from there, drove formation of filopodia through gradual convergence of these lamellipodial filaments into bundles. These data demonstrate a strong relationship between structurally different actin filament arrays and molecular machineries involved in their formation.

## Abstract 3

Formin mDia2 was believed to function mainly in the generation of filopodia in migrating cells. We unexpectedly found that mDia2 is also important for lamellipodia and induces filopodia in association with lamellipodia.

## Introduction

Cell motility is a cyclic process consisting of protrusion of the leading edge followed by retraction of the rear. Actin filament polymerization provides a driving force for protrusion, whereas the shape and dynamics of protrusive organelles depend on the spatial organization of underlying filaments and activity of accessory molecules. Spatially restricted and temporally controlled actin filament nucleation is critical for generating membrane protrusions. Arp2/3 complex and formins are two major actin filament nucleators acting as convergent nodes of signaling pathways leading to initiation of actin-based motility (reviewed in[1,2]. Arp2/3 complex nucleates branched actin filaments [3,4]. Conversely, formins nucleate single linear filaments, binding to and protecting from capping their growing barbed ends [5–7]. Therefore, Arp2/3 complex and formins are thought to have nonoverlapping functions in cells in the formation of dendritic networks and linear bundles, respectively. These distinct roles have been clearly demonstrated in yeast [8], but appear to be conserved also in mammals. Thus, Arp2/3 complex is a key nucleator during lamellipodia extension [9–12] and endocytosis, where it functions downstream of WAVE [13] and N-WASP [14] Arp2/3 activators, respectively. Formins mDia1 and mDia2, instead, play a role in stress-fiber formation [15] and filopodia [16,17], respectively. *Dictyostelium* dDia2 is also both necessary and sufficient for filopodia extension [18].

The concept of functional separation of Arp2/3 complex and formins during leading-edge protrusion to generate dendritic networks in lamellipodia and parallel bundles in filopodia, respectively, is challenged by the observation that filopodia may arise by reorganization of the lamellipodial network [10,19,20]. In this process, termed the convergent elongation, Arp2/3-dependent nucleation was proposed to supply filaments for filopodial bundles. However, other studies point to a nonessential role of Arp2/3 complex in filopodia [12,21] favoring an alternative model whereby formin mDia2 is sufficient to generate a filopodial bundle not necessarily associated with lamellipodia [17,22]. Thus, the mechanisms of filopodia formation and contribution of different actin nucleators to the generation of protrusions in mammalian cells are far from being elucidated.

The domain organization of two related formins, mDia1 and mDia2, and functions of individual domains are well characterized [23,24]. The functional module consisting of FH1 and FH2 domains is responsible for nucleation, barbed-end binding, and anticapping protection of formins in vitro [5–7]. In cells, the FH1FH2 domain of mDia1 can travel on polymerizing barbed ends over significant distances until it stops at the cell membrane [25]. The N-terminal regulatory region upstream of the FH1FH2 module contains the GTPase-binding domain (GBD), the Diaphanous inhibitory domain (DID), the dimerization domain (DD), and the coiled coil region (CC), whereas the Diaphanous autoinhibitory domain (DAD) is present at the C-terminus of the molecule. Full-length Diaphanous-related formins are autoinhibited through intramolecular interaction between DID and DAD, which is thought to be relieved by specific small GTPases binding to GBD and displacing DAD from DID [26]. Disruption of this intramolecular interaction by deleting DAD or DID sequences generates constitutively active mutants of these formins [27].

In this study, aiming to characterize the mDia2-dependent mechanism of filopodia formation, we investigated a role of mDia2 in the leading-edge protrusion by loss-of-function and gain-of-function approaches followed by detailed analyses of the phenotypes using light and electron microscopy (EM) and a combination of both. Unexpectedly, we found that mDia2 was implicated in lamellipodia formation, suggesting that the Arp2/3 complex, despite being essential, was not sufficient for this function. Furthermore, the constitutively active mDia2 mutant efficiently induced filopodia, but these filopodia were formed, not from a focal spot away from lamellipodia, but by the gradual reorganization of lamellipodial filaments converging into filopodial bundles. We also found that specific targeting to the membrane was essential for mDia2 functions in protrusion. The targeting required a multifunctional scaffolding protein Abi1, which also participates in other actin-remodeling events, including regulation of Arp2/3-dependent nucleation [13]. Thus, two types of actin-based protrusions, lamellipodia and filopodia, despite containing structurally distinct actin filament arrays, display considerable dynamic relationship with each other and share mDia2 as a key molecular player.

## Results

### Knockdown of mDia2 by siRNA Inhibits Lamellipodia and Filopodia

**Figure 1:**
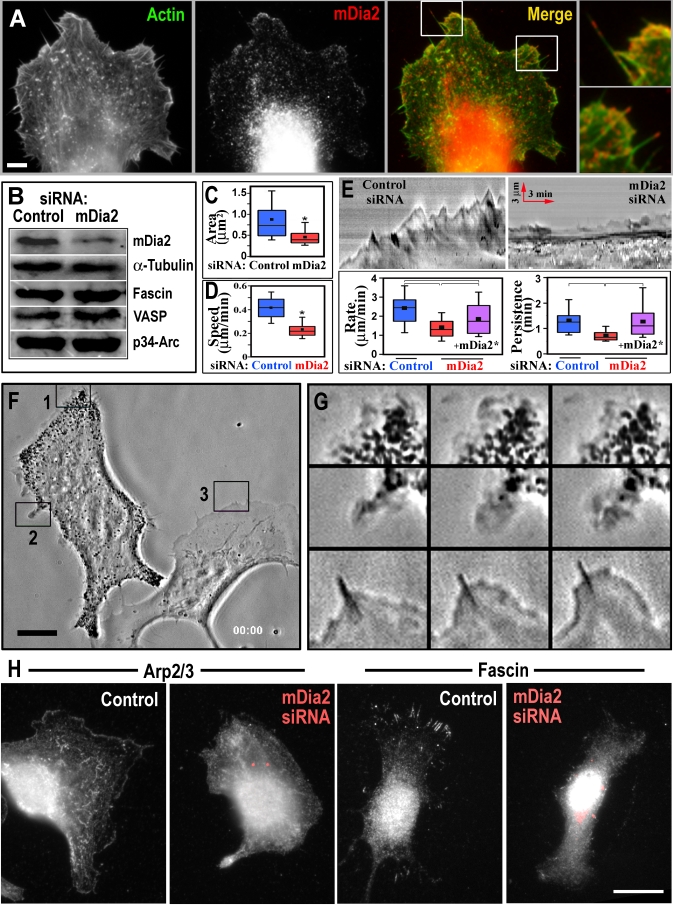
Depletion of mDia2 in B16F1 Cells by siRNA (A) Staining of endogenous mDia2 by antibody (red) and actin by phalloidin (green). mDia2 is concentrated in lamellipodia and at filopodial tips (see insets at right). (B) Western blotting of cell populations transfected with control or mDia2 siRNA. Tubulin is used as a loading control. Amount of mDia2 is decreased by approximately 60%; amounts of other proteins involved in protrusion do not decrease. (C) Inhibition of cell spreading by mDia2 siRNA. Projected cell area was determined 2 h after cell plating. Top and bottom of a box indicate 75th and 25th quartiles, respectively; whiskers indicate 10th and 90th percentiles, respectively; dot is the mean; and the middle line is the median. An asterisk (*) indicates statistical significance (*p* < 0.0001, *n* = 120–129 cells). (D) Inhibition of cell migration by mDia2 siRNA. An asterisk (*) indicates statistical significance (*p* < 0.001, *n* = 34–35 cells). (E) Inhibition of lamellipodia protrusion by mDia2 siRNA. Analysis of kymographs (top) showed that rate and persistence of lamellipodia (bottom) are reduced in mDia2 siRNA-treated cells (red) compared to control siRNA (blue). Coexpression of siRNA-resistant GFP-mDia2* (purple) partially rescues the rate and fully rescues the persistence of mDia2 siRNA cells. Differences between datasets connected by brackets are statistically significant (*p* < 0.001, *n* = 20–36 cells). Red arrows in kymographs indicate the direction and the scale of time and distance. (F) Cell transfected with mDia2 siRNA (left), in contrast to control cell (right), poorly forms lamellipodia. Numbered boxes indicate regions for the time-lapse sequences shown in (G). (G) Frames from time-lapse sequence (25 s apart) for boxed regions in (F) (top, box 1 is shown; middle, box 2; and bottom, box 3). (H) Immunostaining of p16-Arc subunit of Arp2/3 complex (left) and fascin (right) in control and mDia2 siRNA-treated cells. Both proteins lose their characteristic enrichment in lamellipodia and filopodia, respectively. mDia2 siRNA is shown in red. Scale bars indicate 10 μm in (A); 10 μm in (F); and 25 μm in (H).

Mouse melanoma B16F1 cells form broad lamellipodia interspersed by a varying number of filopodia (Figure 1A). During their lifetime, filopodial bundles might remain within lamellipodia and are often termed microspikes, or protrude beyond the leading edge, becoming bona fide filopodia [19]. We determined the intracellular localization of endogenous mDia2 by immunostaining, and found that it concentrated at filopodial tips, but also was distinctly enriched in lamellipodia (Figure 1A). Filopodial localization is consistent with previously shown targeting of activated mDia2 [16,17] and *Dictyostelium* formin dDia2 [18] to filopodial tips, but lamellipodial enrichment was unexpected, and suggested that mDia2 might function in both types of protrusions.

**Figure 2:**
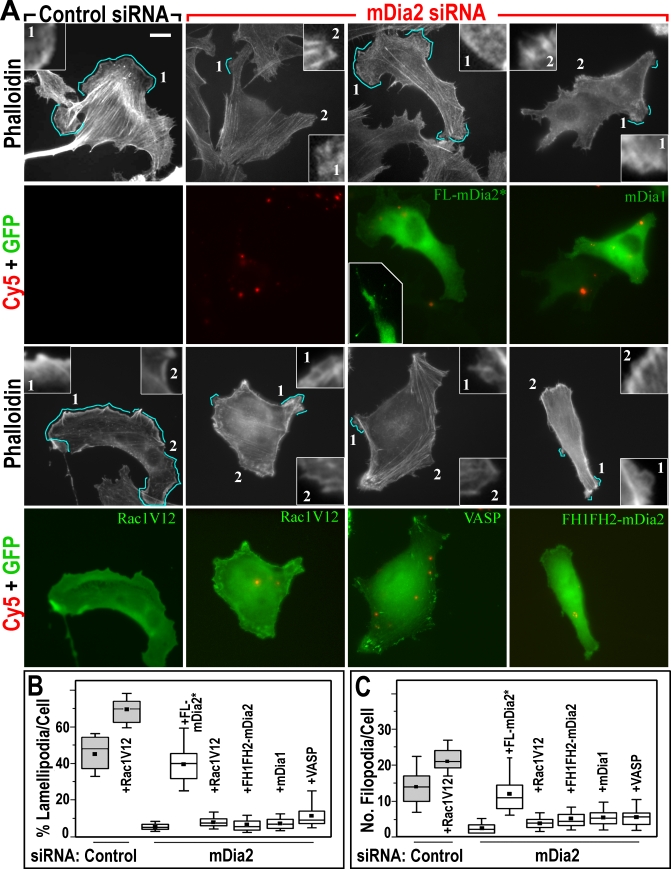
Rescue of mDia2 siRNA Phenotype (A) Phalloidin staining (white) in cells transfected, as indicated, with control or mDia2 siRNA (red) and cotransfected with siRNA-resistant GFP-FL-mDia2*, GFP-mDia1, GFP-Rac1V12, GFP-VASP, or GFP-FH1FH2-mDia2 (green), as indicated. Lamellipodia formation is inhibited by mDia2, but not by control siRNA, and can be rescued by GFP-FL-mDia2*, but not by other proteins. Cyan lines mark examples of edges scored as lamellipodia during quantification shown in (B). Insets in phalloidin channels show close-ups of cell edges scored as positive (1) of negative (2) for lamellipodia presence according to the criteria described in Materials and Methods. Inset in GFP-FL-mDia2 panel shows FL-mDia2* at filopodial tips in another cell. (B and C) Quantification of lamellipodia (B) and filopodia (C) expression in conditions shown in (A). Box-and-whisker plots are as in Figure 1. All differences are statistically significant. *n* = 21–38 cells. Scale bars indicate 10 μm.

We investigated mDia2 functions in cells using the RNA interference (RNAi) approach. Transfection of B16F1 cells with mDia2 short interfering RNA (siRNA) significantly depleted endogenous mDia2, but not other proteins involved in actin-based protrusion (Figure 1B), such as the p34-Arc subunit of Arp2/3 complex, a filopodial actin-bundling protein fascin [28], or VASP, an actin-binding protein localizing to filopodia tips, lamellipodial edges, and focal adhesions [29]. Depletion of mDia2 caused a significant delay in cell spreading (Figure 1C), cell migration (Figure 1D), and a striking inhibition of both lamellipodia and filopodia, as compared to control, scrambled siRNA-treated cells (Figure 1E–1H; Videos S1 and S2). Quantitative analysis of phalloidin-stained cells (Figure 2B) revealed that less than 10% of the cell periphery in mDia2-depleted cells was occupied by actin-rich fringes morphologically resembling lamellipodia (see Materials and Methods for quantification criteria), as compared to approximately 50% in control cells. Furthermore, the presence of lamellipodia sharply declined with decreasing levels of mDia2 (Figure S1). Accordingly, the lamellipodial markers, p16-Arc (Arp2/3 subunit) (Figure 1H), capping protein, Abi1, or VASP (unpublished data) were no longer enriched at the cell edge in mDia2 siRNA-treated cells. Thus, control cells had 53.1 ± 11.1% (*n* = 11) of their perimeter stained with p16-Arc antibody, whereas mDia2 knockdown cells had only 4.4 ± 1.6% (*n* = 10) of Arp2/3-positive edges. Notably, the remaining lamellipodia in mDia2-depleted cells were abnormal morphologically, as they were very narrow in the direction perpendicular to the edge and often resembled small ruffles, whereas classical broad and flat lamellipodia were not observed. These lamellipodia were also abnormal kinetically, as they quickly switched to retraction after transient protrusion (Figure 1G), and their protrusion rate and persistence were significantly reduced (Figure 1E).

Some filopodia also remained after transfection with mDia2 siRNA, but they appeared less rigid than normal, frequently bending and protruding in a curvy path. Although fascin was present in these filopodia, the number of fascin-containing structures was significantly decreased (Figure 1H). No obvious changes in stress-fiber levels or organization were detected (Figure 2A). Depletion of mDia2 also caused accumulation of pigment granules, consistent with a role of mDia2 either in membrane trafficking [30,31] or transcription [32].

The effects of mDia2 siRNA on lamellipodia and filopodia were specific because they could be rescued by RNAi-resistant full-length green fluorescent protein (GFP)-mDia2 (FL-mDia2*) (Figures 1E, 2, and S2). Although GFP-FL-mDia2* localized mainly cytoplasmically, as reported earlier for wild-type FL-mDia2 [27], it was sometimes enriched in lamellipodia and at filopodial tips, especially in rescued cells (Figure 2A), apparently because of less competition from the endogenous untagged protein. As additional specificity tests, we attempted to rescue the mDia2 knockdown phenotype by expressing proteins with potentially redundant activities, a closely related formin GFP-mDia1 and GFP-VASP [33,34]. However, neither of them produced a significant rescue, suggesting nonredundant functions with mDia2. To test a possibility that inhibition of lamellipodia involved down-regulation of Rac1, we expressed constitutively active Rac1V12 in mDia2 knockdown cells. Although Rac1V12 increased lamellipodia and filopodia in control cells, it did not restore their amount in mDia2 siRNA-treated cells. Interestingly, FH1FH2-mDia2 also did not rescue the phenotype (see below).

**Figure 3:**
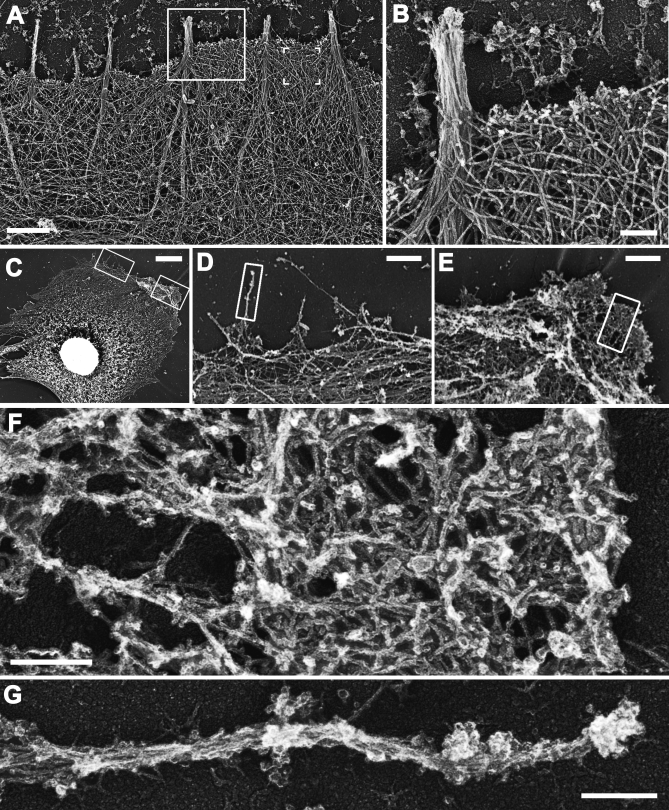
EM of Protrusions in Control (A and B) and mDia2-Depleted (C–G) B16F1 Cells (A and B) Overview of an edge (A) and enlargement of the boxed region (B) of untreated cell show dense actin filament network in lamellipodia and parallel bundles in filopodia. Filaments in filopodia roots splay apart and merge with the surrounding network (A). Brackets in (A) mark a region analyzed in Figure 5E. (C) Overview of mDia2-depleted cell. Boxes indicate the magnified regions shown in (D) and (E). (D and E) Intermediate magnification of boxed regions in (C). Boxes indicate the magnified regions shown in (F) and (G). (F and G) High magnification of boxed regions in (E) and (D), respectively. Note overall inhibition of protrusions (C–E) and abnormal organization of remaining lamellipodia (F) and filopodia (G). Scale bars indicate 1 μm in (A), (D), and (E); 0.2 μm in (B), (F), and (G); and 5 μm in (C).

Platinum replica EM was used to reveal specific defects caused by mDia2 depletion in the architecture of the protrusive organelles (Figure 3). Normal lamellipodia in B16F1 cells are filled with branched actin network consisting of combination of long and short filaments, whereas filopodia contain tight bundles of long actin filaments (Figure 3A and 3B) [4,19]. In mDia2-knockdown cells, actin filament organization at the cell periphery was severely disrupted (Figure 3C–3G). Consistent with light microscopic data, only a small fraction (8.7 ± 8.9%, *n* = 5) of cell perimeter in these cells contained dendritic actin network characteristic for lamellipodia. The rare remaining lamellipodia contained patches of dense network poorly connected to the rest of the cytoskeleton and formed by short branching actin filaments (Figure 3F), while long filaments were not obvious there. This morphology suggests that Arp2/3-dependent actin nucleation likely remains functional [4], but anticapping protection and/or linear filament elongation was compromised [33]. Remaining filopodia, instead of a regular bundle of long, parallel filaments, contained less-uniform actin arrays (Figure 3G).

Collectively, these results reveal a novel role of formin mDia2 in the formation of lamellipodia, likely involving facilitated formation of long filaments, and corroborate its function in filopodial protrusion [16–18] by an alternative approach.

### Effects of Constitutively Active ΔGBD-mDia2 on Lamellipodia Structure and Dynamics

In addition to loss-of-function analysis by RNAi, we also tested mDia2 functions by a gain-of-function approach using a constitutively active mDia2 mutant (ΔGBD-mDia2) lacking GBD and a part of DID [15,16]. We reasoned that any unusual features induced by this mutant would point to specific molecular mechanisms of mDia2 involvement in cell protrusion.

**Figure 4:**
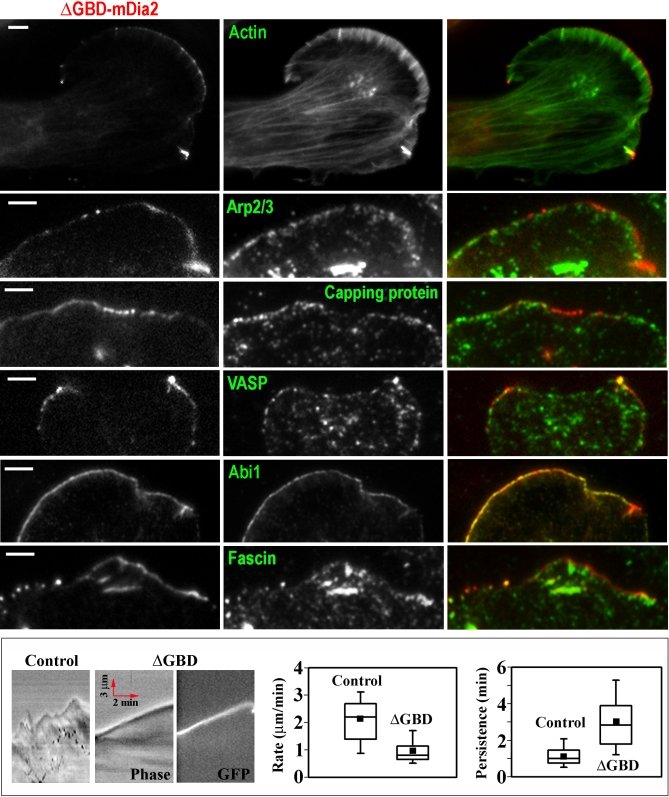
Lamellipodia of ΔGBD-mDia2–Expressing B16F1 Cells (A) Distribution of GFP-ΔGBD-mDia2 (red) and indicated cytoskeletal proteins (green) detected by phalloidin (Actin) or immunostaining (all others). ΔGBD-mDia2–positive lamellipodia contain normal lamellipodial components: actin, Arp2/3 complex, capping protein, VASP, Abi1, and a filopodial marker, fascin. Scale bars indicate 2.5 μm. (B) Lamellipodia dynamics in control and ΔGBD-positive lamellipodia. Left: kymographs; right: quantification of the rate and persistence of lamellipodial protrusion. ΔGBD-positive lamellipodia are slower, but remarkably persistent, as compared to control cells (*n* = 23–39 cells, *p* < 0.0001). Red arrows in kymographs indicate the direction and the scale of time and distance.

**Figure 5:**
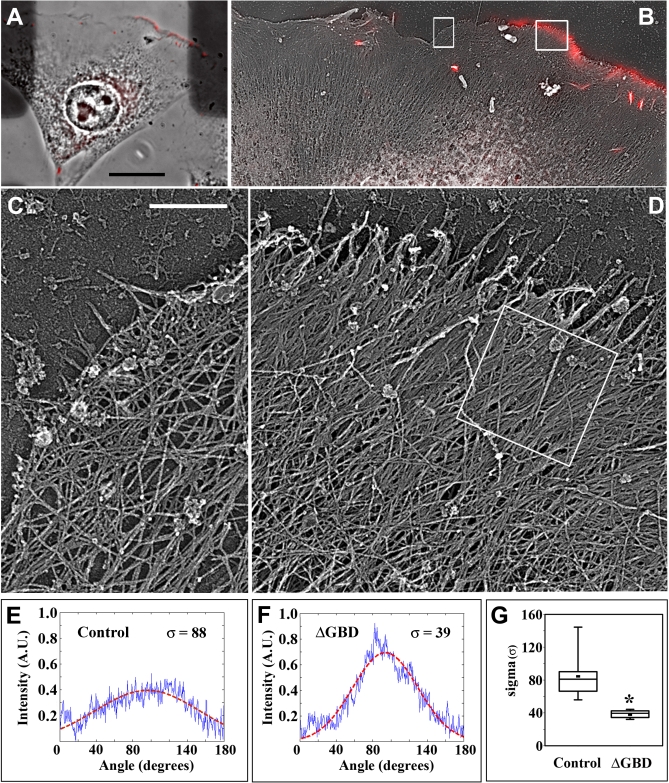
Structural Organization of Lamellipodia in ΔGBD-mDia2–Expressing Cells Correlative EM is used to unambiguously identify ΔGBD-mDia–positive regions. (A) Phase contrast image overlaid with the GFP-ΔGBD-mDia2 image in red. (B) Low-magnification EM image of the periphery of the same cell overlaid with GFP image in red. Boxes indicate the magnified regions shown in (C) and (D). (C and D) Enlarged boxed regions from (B). (C) ΔGBD-mDia2–negative lamellipodium containing branching actin network. (D) Lamellipodium with continuous GFP fluorescence is dominated by densely packed, long parallel filaments. Box indicates a region analyzed in Figure 5F. Scale bars indicate 10 μm in (A) and in 0.5 μm (C) and (D). (E–G) Actin filament orientation in control and ΔGBD-mDia2–positive lamellipodia. (E and F) Plots of radial intensity versus angle (blue) of Fourier transforms generated from the ΔGBD-mDia2–positive lamellipodium in the box in (D) (E), and from the control lamellipodium indicated by brackets in Figure 3A (F). Standard deviation (σ) of Gaussian fits (red), which is used as a parameter of mutual filament orientation, is shown in respective plots. (G) Distributions of σ for ΔGBD-mDia2-positive and control lamellipodia are significantly different (*p* < 0.0001; *n* = 8–9 cells).

**Figure 6:**
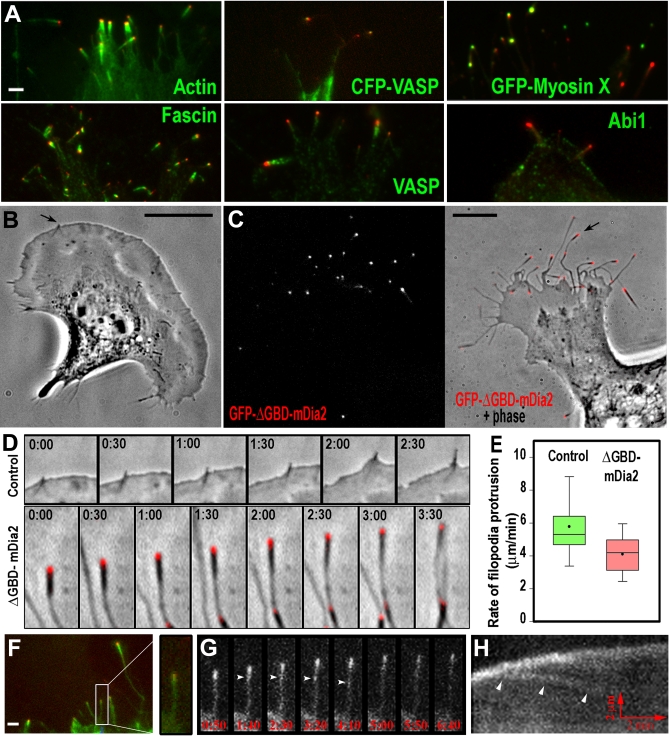
Filopodia Induction by ΔGBD-mDia2 (A) Cytoskeletal proteins in ΔGBD-induced filopodia. GFP-, YFP-, or mRFP1-ΔGBD-mDia2 (red)-induced filopodia contain filopodial markers, actin, fascin, VASP, and myosin X, but Abi1 is not enriched there. Fascin, which normally localizes throughout the filopodia, is present only in thick distal domains. VASP localizes to filopodia tips (panel CFP-VASP) or shafts (VASP) in cells expressing low and high levels of ΔGBD-mDia2, respectively. Myosin X at the filopodial tips either colocalizes with or is more distal than GFP-ΔGBD-mDia2. Indicated proteins were visualized by coexpressing fusion proteins (panels CFP-VASP and GFP-myosin X), phalloidin staining (Actin), or immunostaining (all others). (B) Control B16F1 cell contains relatively short filopodia partially or completely embedded into lamellipodia. Arrow indicates a filopodium shown in (D). (C) Cell expressing GFP-ΔGBD-mDia2 has long, curvy filopodia with ΔGBD-mDia2 at their tips. GFP fluorescence alone (left) and phase overlaid with GFP image in red (right). Arrow indicates a filopodium shown in (D). (D) Filopodia dynamics. Frames from time-lapse sequences showing protrusion of control filopodium (top) and ΔGBD-mDia2–induced club-like filopodium (bottom) indicated by arrows in (B) and (C), respectively. Time shown in minutes:seconds. (E) Protrusion rates of control (*n* = 48) and ΔGBD-mDia2–induced (*n* = 62) filopodia. Box-and-whisker plots are as in Figure 1. The difference is statistically significant at *p* < 0.001. (F–H) Dynamics of GFP-actin in mRFP-ΔGBD-mDia2–expressing cell. Overview (F) shows filopodia having actin (green) enriched in distal segments and mRFP-ΔGBD-mDia2 (red) localizing at their tips. Boxed filopodium is enlarged at right. Time-lapse sequence (G) and kymograph (H) of the boxedfilopodium from (F) shows retrograde movement and disappearance of actin speckles (arrowheads) while the filopodium protrudes. Scale bars indicate 2.5 μm in (A); 10 μm in (B) and (C); 1 μm in (D); or 2.5 μm in (F).

In B16F1 cells, GFP-ΔGBD-mDia2 localized to a subset of lamellipodia (Figures 4A and 5) and to filopodial tips (Figure 6) with relatively low cytoplasmic fluorescence. This localization is consistent with the functional importance of the endogenous protein for these protrusions. Compared to endogenous mDia2, ΔGBD-mDia2 had narrower distribution at the very edge of lamellipodia. ΔGBD-mDia2–positive lamellipodia contained normal lamellipodial components, Arp2/3 complex, capping protein, VASP, and Abi1 (Figure 4A). However, at edges with high levels of ΔGBD-mDia2, capping protein was not detected, possibly reflecting anticapping activity of formins [7]. Conversely, the pattern of ΔGBD-mDia2 at the leading edge frequently correlated with that of Abi1. However, Abi1 only partially colocalized with the endogenous mDia2, which formed a broader band at the leading edge (Figure S3). An unusual feature of ΔGBD-mDia2–positive lamellipodia was the presence of fascin (Figure 4A), which is normally not detected in B16F1 lamellipodia [19]. Analysis of dynamic behavior of ΔGBD-positive lamellipodia revealed that their protrusion is remarkably persistent, whereas the rate of protrusion is slower than that of control cells (Figure 4B).

Correlative EM analysis ΔGBD-mDia2–positive lamellipodia (Figure 5) revealed that they had unusual abundance of long, parallel, unbranched actin filaments (Figure 5D). Quantitative analysis of filament orientation revealed a narrow distribution of angles in ΔGBD-mDia2–positive lamellipodia compared to very broad distribution in control lamellipodia (Figure 5E–5G). Enrichment in long filaments after expression of constitutively active mDia2 complements the siRNA data showing apparent filament shortening after mDia2 depletion. These long filaments frequently displayed apparently free (not engaged in branch formation) proximal ends (Figure S4), which are very rare in normal lamellipodia [35], suggesting that active mDia2 might nucleate these linear filaments within lamellipodia. However, ΔGBD-mDia2–positive lamellipodia also contained some branched filaments (Figure S4) and Arp2/3 complex (Figure 4A), suggesting that the lamellipodial network represents a mixture of linear and dendritic actin filament arrays. The atypical abundance of long, parallel filaments induced by constitutively active mDia2 might cause the peculiar recruitment of fascin.

Thus, the gain-of-function approach showed that the constitutively active mDia2 mutant was recruited to lamellipodia and induced structural reorganization of the lamellipodial actin network by promoting formation of long filaments, as well as increased the persistence of lamellipodia protrusion. These results corroborate the findings of mDia2 knockdown experiments, which suggested mDia2 function in lamellipodia.

### Constitutively Active ΔGBD-mDia2 Induces Club-Like Filopodia

Our findings of mDia2 involvement in lamellipodia formation apparently contrast with previous reports showing a role of mDia2 [16,17] and *Dictyostelium* dDia2 [18] in filopodia. However, we found that the lamellipodial localization of GFP-ΔGBD-mDia2 was transient and especially evident at early stages of transfection. At later times, ΔGBD-mDia2 induced abundant filopodia with ΔGBD-mDia2 localizing at their tips (Figure 6; Video S3), consistent with other reports [16–18]. We noticed, however, that these filopodia frequently had an unusual club-like shape with a thick actin-rich distal domain on a thin stalk (Figure 6A, 6C, and 6D). Nonetheless, they contained conventional filopodial markers: fascin, VASP, and myosin X [36], albeit with a somewhat altered localization (Figure 6A). Fascin was concentrated predominantly in thick distal parts of filopodia. VASP colocalized with ΔGBD-mDia2 at filopodial tips in some cells, but high levels of ΔGBD-mDia2 seemed to displace VASP from tips to the filopodial shafts, possibly reflecting VASP's bundling ability [37,38]. Abi1, which usually localizes to lamellipodial edges and filopodial tips [39], was faint at the filopodial tips enriched in ΔGBD-mDia2.

Filopodia induced by ΔGBD-mDia2 frequently were very long, flexible, not attached to the substratum, and occasionally appeared on the dorsal cell surface (Figure S5), resembling filopodia induced by a small GTPase Rif through mDia2 [17]. During protrusion, ΔGBD-mDia2 remained at filopodial tips (Figure 6D), consistent with the formins' ability to ride on elongating barbed ends [25]. The thick termini of filopodia moved forward, remaining approximately of the same length, suggesting actin filament treadmilling within these domains (Figure 6D). Consistent with this idea, GFP-actin speckles moved retrogradely in ΔGBD-mDia2–induced filopodia and disappeared upon or after exiting the thick terminal domains (Figure 6F–6H; Video S4). The protrusion rate of ΔGBD-mDia2–positive filopodia was slightly lower than that of control filopodia (Figure 6E).

**Figure 7:**
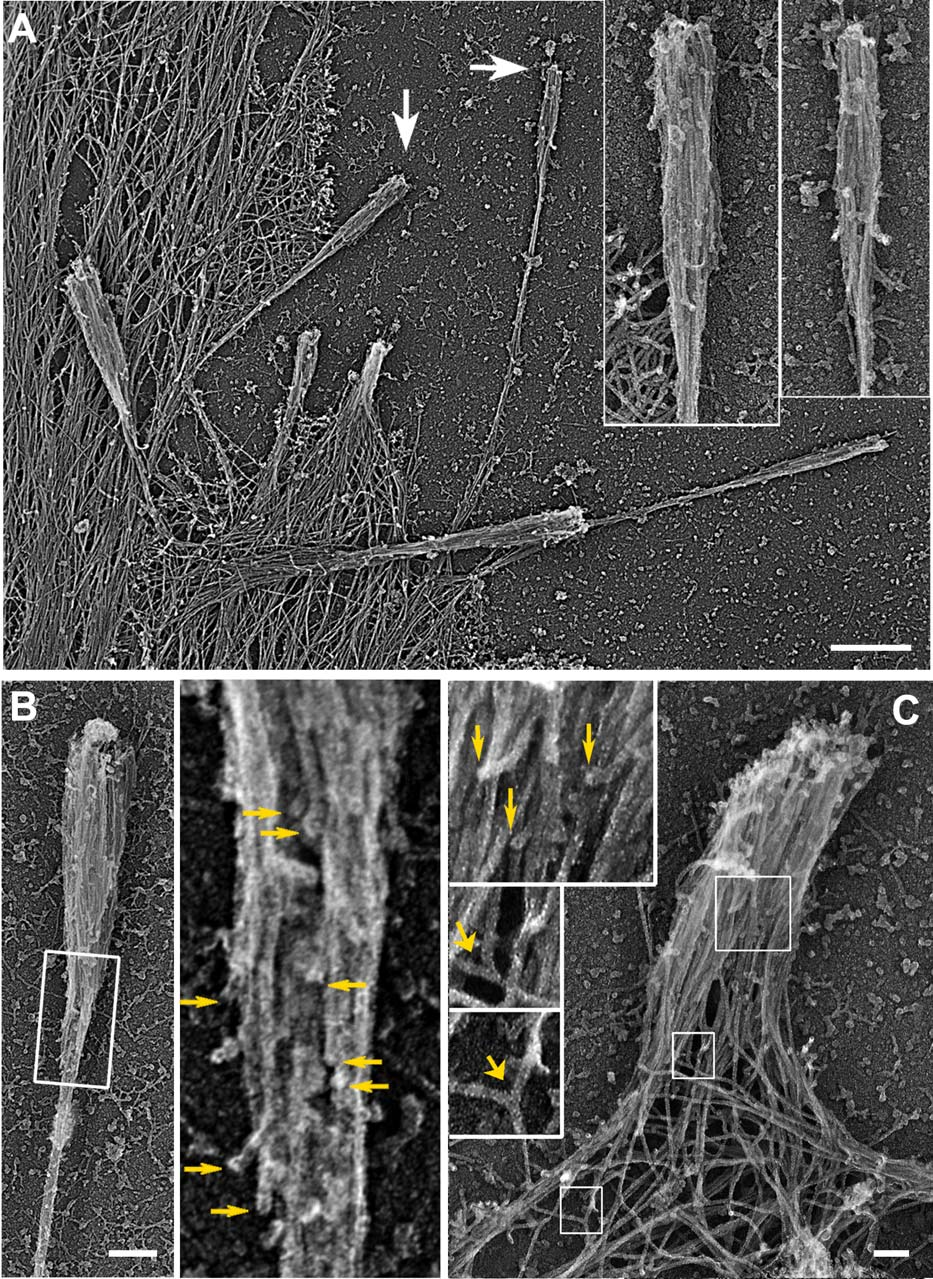
EM of ΔGBD-mDia2–Positive Filopodia (A) Overview of a peripheral region of ΔGBD-expressing cell. Arrows point to filopodia enlarged in insets. (B) Club-like filopodium induced by ΔGBD-mDia2. Numerous filaments present in the thick terminal bundles are gradually lost as the bundle tapers towards the rear (bottom of the image). Boxed region in left panel is enlarged at right, and unbound filament ends are marked by arrows. (C) Filopodium in ΔGBD-mDia2–expressing cell contains filaments originating from branch points (lower insets, wide arrows) and filaments having unbound “pointed” ends (upper inset, narrow arrows). Scale bars indicate 0.5 μm in (A); 0.2 μm on (B); or 0.1 μm in (C).

By EM analysis, ΔGBD-mDia2–mediated filopodia contained actin bundles, which looked normal in their distal domains, but displayed informative differences in the proximal regions (Figure 7). In normal filopodia (Figure 3A), filaments in their roots commonly splay apart, reflecting the convergent elongation process of filopodia formation, and frequently terminate at branch points in the surrounding network [19]. Conversely, many ΔGBD-mDia2–induced filopodia displayed tapered bundles at the rear, with numerous unbound proximal ends (Figure 7A and 7B), which are predicted to be pointed ends based on their orientation. Since actin network is assembled predominantly at the leading edge and then treadmills backwards, undergoing little rearrangement except depolymerization, the structural organization of the network to some extent portrays its immediate history [40]. Therefore, tapered roots of ΔGBD-mDia2–induced bundles suggest that these filaments were nucleated by ΔGBD-mDia2 and/or they were nucleated by Arp2/3 complex, but then debranched and depolymerized from the pointed ends. Notably, some ΔGBD-mDia2–induced filopodia contained bundles with splayed roots (Figure 7A and 7C), and some filaments in these filopodia originated from a branch point (Figure 7C), suggesting that Arp2/3-nucleated filaments might contribute to bundle formation.

Together, these results confirm the previous observations that mDia2 plays a role in filopodia generation, and localizes to filopodial tips. We extend prior observations by showing that although filopodia induced by active mDia2 share many features with normal filopodia, they also display an abnormal abundance of prematurely terminated filaments in the proximal parts of filopodial bundles.

### ΔGBD-mDia2–Induced Filopodia Are Formed from Lamellipodia

**Figure 8:**
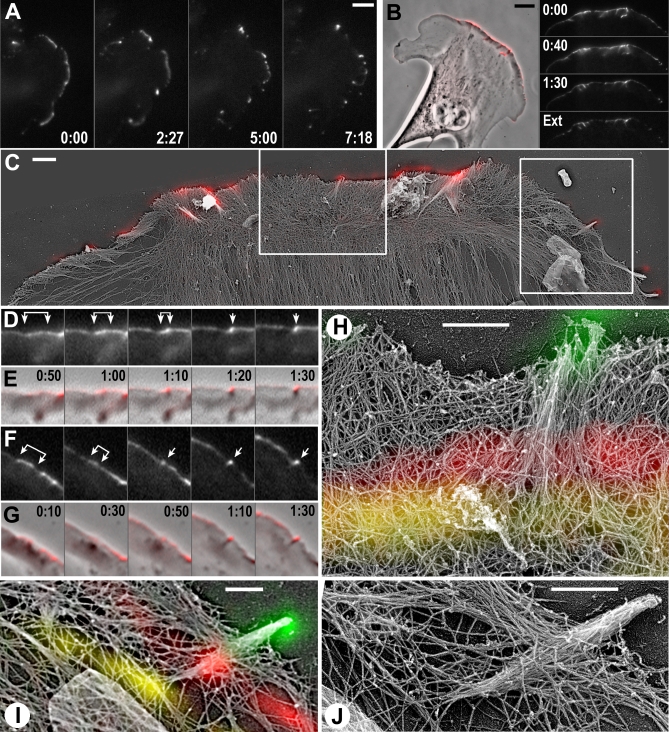
Initiation of ΔGBD-mDia2–Induced Filopodia in B16F1 Cells (A) Time-lapse sequence shows how linear lamellipodial distribution of GFPΔGBD-mDia2 transforms into a series of dots. Time is shown in minutes:seconds. (B–J) Correlative EM of nascent ΔGBD-mDia2–induced filopodia. (B) Phase contrast of ΔGBD-mDia2–expressing cell overlaid with GFP image in red (left) and frames from live GFP sequence including an image after extraction (Ext) (right). Most fluorescence remains after extraction. (C) EM overview of the periphery of the cell shown in (B) overlaid with the GFP image taken after extraction (red). Boxes indicate regions shown in (D) and (E) (left) and in (F) and (G) (right). (D–G) GFP (D) and (F) and phase overlaid with GFP in red (E) and (G) sequences showing formation of nascent filopodia. In both cases, GFP dots at the tips of filopodia (arrows) were formed by condensation of linear lamellipodial fluorescence (brackets). (H–J) EM of nascent filopodia shown in (D) and (E) ([H]) or in (F) and (G) ([I] and [J]). Colors in (H) and (I) represent projected GFP images from corresponding regions taken at those time points: yellow, 0:00; red, 0:50; and green, 1:30. Since ΔGBD-mDia2 is dynamically associated with the advancing cell edge, its localization marks the position of the cell edge at the respective time point. Therefore, only structures behind the color line existed at that time, and structures in front of the line were formed later. In (H), ΔGBD-mDia2 fluorescence at the 0:00 time point (yellow) projects to a region with unbundled filaments behind it; subsequently, filaments begin to converge, and a partially condensed fluorescence (red, 0:50) projects to partially bundled filaments; by the end of the sequence, when GFP condenses into a dot (green), a bundle is formed. In (I), line of ΔGBD-mDia2 in the first frame (yellow) projects to a region with sparse actin filaments, possibly due to network disassembly (see text). Scale bars indicate 5 μm in (A) and (B); 1 μm in (C); or 0.5 μm in (H–J).

We next asked whether mDia2 roles in lamellipodia and filopodia are related. Kinetic analysis of filopodia initiation showed that virtually all ΔGBD-mDia2–induced filopodia formed from ΔGBD-mDia2–positive lamellipodia (Figure 8; Video S5). In this pathway, a line of ΔGBD-mDia2 fluorescence at the lamellipodial leading edge gradually condensed into dots at the tips of newly formed filopodia (Figure 8A), similar to VASP behavior in naturally occurring filopodia [19]. Full-length GFP-mDia2 displayed similar behavior (Video S6), although high cytoplasmic fluorescence of this autoinhibited protein significantly decreased contrast of the leading edge signal. Newly formed ΔGBD-mDia2–induced filopodia might subsequently fuse with each other, generating larger filopodia and gradually acquiring a club-like shape (Figure S5). Some filopodia subsequently translocated to the dorsal surface of lamella; the majority of dorsal protrusions were formed by this pathway (Figure S5).

To understand the mechanism of lamellipodia-to-filopodia transition, we performed correlative EM for cells with known live history (Figure 8B–8J; Video S7–S9). Figure 8 shows two examples of filopodia formed during live imaging. One filopodium began to form approximately 30 s before fixation, as judged from condensation of GFP fluorescence at the leading edge (Figure 8D and 8E). In the corresponding EM image, this filopodium consisted of long filaments originating from a broad area at the base and coming together at the tip (Figure 8H). Projection of the life history onto EM image revealed that filaments converged in parallel with condensation of GFP fluorescence (see Figure 8 legend for detail), similar to normal filopodia [19].

The second example (Figure 8F and 8G) shows a slightly older filopodium formed from a line of GFP fluorescence visible in the first frame of the movie. By approximately 50–60 s before fixation, the line gradually condensed into a dot, suggesting that the filopodial bundle was formed by that time. Subsequently, the dot moved approximately 1 μm as the filopodium protruded. In the corresponding EM image (Figure 8I and 8J), the filopodium contained a spindle-shaped actin bundle that tapered both toward the front (perhaps because of tighter filament bundling) and toward the rear due to gradual termination of filaments. The projection of the fluorescence history of this filopodium onto the EM image showed a sparse actin network proximally from the position of the leading edge at time 0:00 (yellow line in Figure 8I). Such morphology might be explained by nucleation of a filament bundle by ΔGBD-mDia2 at a local spot in actin-poor region, followed by elongation of the bundle, as proposed [22]. However, in this case, we would see an emergence and gradual advance of a dot of ΔGBD-mDia2 fluorescence in the time-lapse movie instead of a fluorescent line converging into a dot (Figure 8F). As we showed above (see Figure 5), the linear distribution of ΔGBD-mDia2, as it appears at the 0:00 time point for this filopodium (Figure 8F and 8I), always associates with dense lamellipodial network of long, aligned filaments. Thus, the tapered shape of the filopodium root among sparse actin network is more consistent with the alternative possibility that actin depolymerization eliminated much actin from the filopodium rear in parallel with the filopodium protrusion at the front.

Correlative analysis of six cells imaged live for 90–120 s showed that all nascent filopodia (31 total) emerged by condensation of linear lamellipodial fluorescence. At EM level, 14 out of these 31 filopodia were similar in structure to the first example in Figure 8; five were similar to the second example; nine displayed an intermediate organization, having a fully formed distal bundle with well-preserved splayed filaments in the root; they looked similar to the filopodium marked by an asterisk in Figure 7; and one filopodium contained converging filaments like in the first example, but showed significant actin depletion at the root, like in the second example.

These data, collectively, indicate that induction of filopodia by active mDia2 temporally and mechanistically follows the mDia2 appearance in lamellipodia. Long, unbranched actin filaments induced by ΔGBD-mDia2 in the lamellipodial network seem to play an important role in this process by expressing a high tendency to converge into bundles, and leading to filopodia induction in association with pre-existing lamellipodia.

### mDia2 Is Specifically Recruited to the Leading Edge to Induce Protrusions

**Figure 9:**
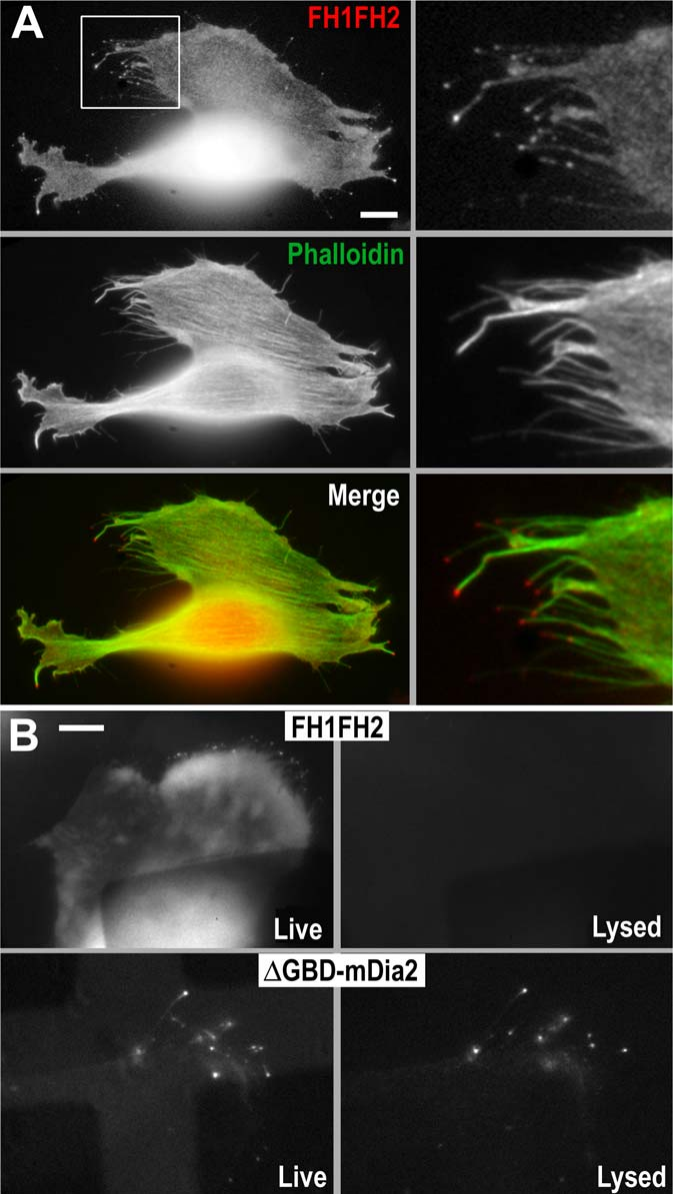
Targeting of mDia2 to the Leading Edge (A) GFP-FH1FH2-mDia2 in B16F1 cells is not efficiently targeted to the membrane and weakly induces filopodia. GFP fluorescence (top) and F-actin (middle) are enriched throughout the cell. Boxed region is enlarged at right. Contrast enhancement shows some FH1FH2-mDia2 at the tips of filopodia. (B) FH1FH2-mDia2, in contrast to ΔGBD-mDia2, is not anchored to the cytoskeleton. Cells expressing GFP-FH1FH2-mDia2 (top) or ΔGBD-mDia2 (bottom) are shown live (left) and detergent-extracted (right) after identical image processing.

FH1FH2 constructs of many formins are sufficient to nucleate actin filaments and bind barbed ends [24], whereas sequences upstream of this module are required for targeting of some formins [41,42]. Enrichment of ΔGBD-mDia2 at the leading edge and filopodial tips (Figures 4 and 6) may be mediated simply by binding to barbed ends through the FH2 domain, or involve an additional targeting mechanism. We tested this idea using FH1FH2-mDia2 mutant (Figure 9).

GFP-FH1FH2-mDia2 was not able to rescue the mDia2 siRNA phenotype (Figure 2) and, when expressed in wild-type B16F1 cells, displayed different behavior as compared to ΔGBD-mDia2 (Figure 9; Video S10): (1) it induced fewer and shorter, evenly thin, but not club-shaped, filopodia; (2) it localized mostly in the cytoplasm and poorly at filopodial tips; and (3), in contrast to ΔGBD-mDia2, GFP-FH1FH2-mDia2 was easily removed by detergent extraction (Figure 9B). Truncation of the FH2 domain from the C-terminus or its complete removal from FH1FH2-mDia2 abrogated filopodia-inducing ability and localization to filopodia tips (Figure S6). Thus, although mDia2 nucleating and barbed end-binding module FH1FH2 is required for and capable of tip localization and weak filopodia induction, additional motifs of ΔGBD-mDia2 are needed for robust mDia2 targeting to the leading edge and efficient induction of filopodia. This is strengthened by the observation that an analogous mutant of mDia1 localized differently (Figure S6). Similar to previous reports [43,44], active mDia1 displayed strong cytoplasmic fluorescence with weak targeting to the edges.

### Abi1 Is Required for ΔGBD-mDia2 Targeting and Efficient Induction of Filopodia

Searching for a molecular mechanism of mDia2 targeting, we concentrated on proteins displaying robust localization to the leading edge of lamellipodia. Ena/VASP proteins were likely not involved, as ΔGBD-mDia2 localized properly in Ena/VASP-deficient MV^D7^ cells (Figure S6). Next, we considered the WAVE/Abi1/Nap1/PIR121/HSP300 (WANP) complex [11,45], a major activator of Arp2/3 complex in lamellipodia, because Abi1 [39] and WAVEs [46] localize robustly to the lamellipodial leading edge. Depletion of any member of WANP complex causes degradation of its other components [11,47]. Thus, we tested whether mDia2 would associates to any of the members of the WANP complex.

**Figure 10:**
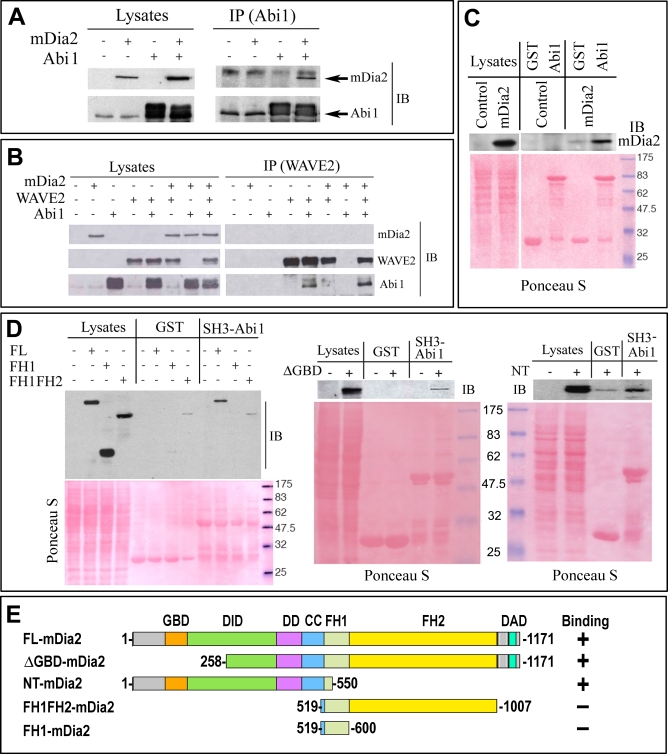
mDia2 Interacts with Abi1 (A) mDia2 and Abi1 coimmunoprecipitate. Total cellular lysates (1 mg) of 293T cells cotransfected with GFP-FL-mDia2 and Abi1, alone or in combination, were immunoprecipitated with an anti-Abi1 antibody. Lysates (20 μg) and immunoprecipitates (IP) were immunoblotted (IB) with the indicated antibodies. Slower migrating Abi1 bands reflect hyperphosphorylation associated with ectopic expression of Abi1 [11,49]. (B) mDia2 and WAVE2 do not coimmunoprecipitate. Total cellular lysates (1 mg) of 293T cells cotransfected with GFP-FL-mDia2, WAVE2, and Abi1, alone or in combination, were immunoprecipitated with an anti-WAVE2 antibody. Lysates (20 μg) and immunoprecipitates (IP) were immunoblotted (IB) with the indicated antibodies. (C) Recombinant full-length Abi1 binds FL-mDia2. Total cellular lysates (1 mg) of 293T cells, untransfected (control) or transfected with GFP-FL-mDia2 (mDia2), were incubated with 1.5 μM of immobilized GST-Abi1 or GST. Lysates (20 μg) and bound proteins were resolved by SDS-PAGE, stained with Ponceau S to detect GST-fusion proteins, and immunoblotted (IB) with the indicated antibodies. (D) The SH3 domain-containing fragment of Abi1 mediates the interaction with mDia2. Total cellular lysates (1 mg) of 293T cells, untransfected or transfected with GFP-FL-mDia2 (FL), GFP-FH1-mDia2 (FH1), GFP-FH1FH2-mDia2 (FH1FH2), GFP-ΔGBD-mDia2 (ΔGBD), or GFP-N-terminal fragment of mDia2 (NT) were incubated with 1.5 μM immobilized GST-Abi1-SH3 (SH3-Abi1; aa 330–480) or GST. Lysates (20 μg) and bound proteins were resolved by SDS-PAGE, stained with Ponceau S to detect GST-fusion proteins and immunoblotted (IB) with the indicated antibodies. Molecular weight markers in (C) and (D) are shown in blue. (E) Summary of binding activities of mDia2 constructs to SH3-Abi1.

Ectopically coexpressed GFP-FL-mDia2 and Abi1 (Figure 10A), but not WAVE (Figure 10B), Nap1, or PIR121 (unpublished data), readily coimmunoprecipitated. Furthermore, recombinantly produced full-length Abi1 associated in vitro with FL-mDia2 (Figure 10C). Finally, a C-terminal Abi1 fragment containing the SH3 domain was able to associate in in vitro binding assays with FL-mDia2, ΔGBD-mDia2, and an N-terminal fragment of mDia2 containing residues up to the beginning of the FH1 domain, but not with FH1FH2-mDia2 or FH1-mDia2 (Figure 10D and 10E). These data map the Abi1-interacting region on mDia2 to amino acids 258–518 within the N-terminal regulatory region (part of DID + DD + CC). Lack of interaction with FH1FH2-mDia2 and FH1-mDia2 argues against a possibility of nonspecific interaction between the proline-rich FH1 domain of mDia2 and SH3 of Abi1.

**Figure 11:**
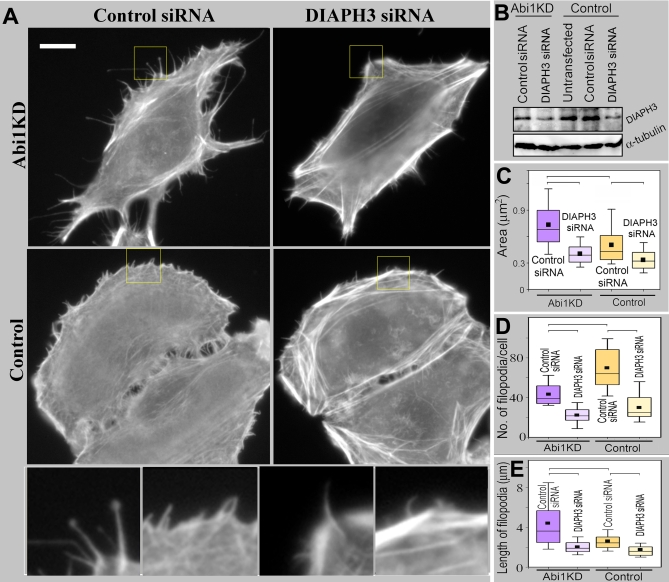
Roles of Abi1 and DIAPH3 in Protrusion (A) Inhibition of protrusions by DIAPH3 siRNA (phalloidin staining). Boxed regions are shown enlarged at the bottom of the panel. (B) Western blot of Abi1KD and control HeLa cells after transfection with control or DIAPH3 siRNA. Abi1KD cells express 75% DIAPH3 compared to control HeLa cells. DIAPH3 siRNA depleted 80% and 55% of DIAPH3 in control and Abi1KD cells, respectively. (C) Inhibition of spreading of control and Abi1KD cells by DIAPH3 siRNA. Projected cell area is determined 2 h after cell plating. Differences between datasets connected by brackets are statistically significant (*p* < 0.0001, *n* = 130–252 cells). (D) and (E) Filopodia number (D) and length (E) in Abi1KD and control cells after transfection with control or DIAPH3 siRNA. Box-and-whisker plots are as in Figure 1. Differences between datasets connected by brackets are statistically significant (*p* < 0.001, *n* = 265–1,102 filopodia from 11–31 cells). Scale bars indicate 10 μm.

To assess the physiological relevance of this interaction, we used a previously characterized stable line of Abi1-RNAi–interfered (Abi1KD) HeLa cells with approximately 90% depletion of Abi1 (Figure S7), which fail to form lamellipodia in response to a variety of stimuli, but can spread and form filopodia [11]. We first examined filopodia in Abi1KD cells quantitatively and by EM (Figures 11 and S7). Control HeLa cells formed a mixture of filopodia and small lamellipodia or ruffles, whereas Abi1KD cells lacked ruffles and displayed long filopodia (Figures 11A and S7). Since filopodial bundles are normally partially embedded into a lamellipodial actin network, inhibition of lamellipodia likely exposed these internal parts, leading to apparent elongation of filopodia. Despite having longer filopodia, Abi1KD cells had fewer filopodia per cell than control cells (Figure 11A and 11D), indicating that Abi1 and/or its interacting partners, such as other members of WANP complex, may play a role in filopodia formation. Interestingly, despite almost complete absence of a lamellipodial dendritic network, EM analysis revealed multiple branched filaments in the roots of filopodia in Abi1KD cells (Figure S7), suggesting a preferential formation of filopodia at the sites with residual activity of Arp2/3 complex.

We tested next whether the human ortholog of mDia2 (DIAPH3) plays a role in filopodia formation in control and Abi1KD HeLa cells using both loss-of-function and gain-of-function approaches. Similar to B16F1 cells, DIAPH3 siRNA significantly depleted DIAPH3 expression and impaired cell spreading and protrusive activity in control and Abi1KD HeLa cells (Figure 11). DIAPH3 depletion decreased filopodia length and number in Abi1KD HeLa cells, and inhibited ruffles and filopodia in control HeLa cells, confirming in the human system that DIAPH3 is an important part of the mechanism of lamellipodia and filopodia formation.

**Figure 12:**
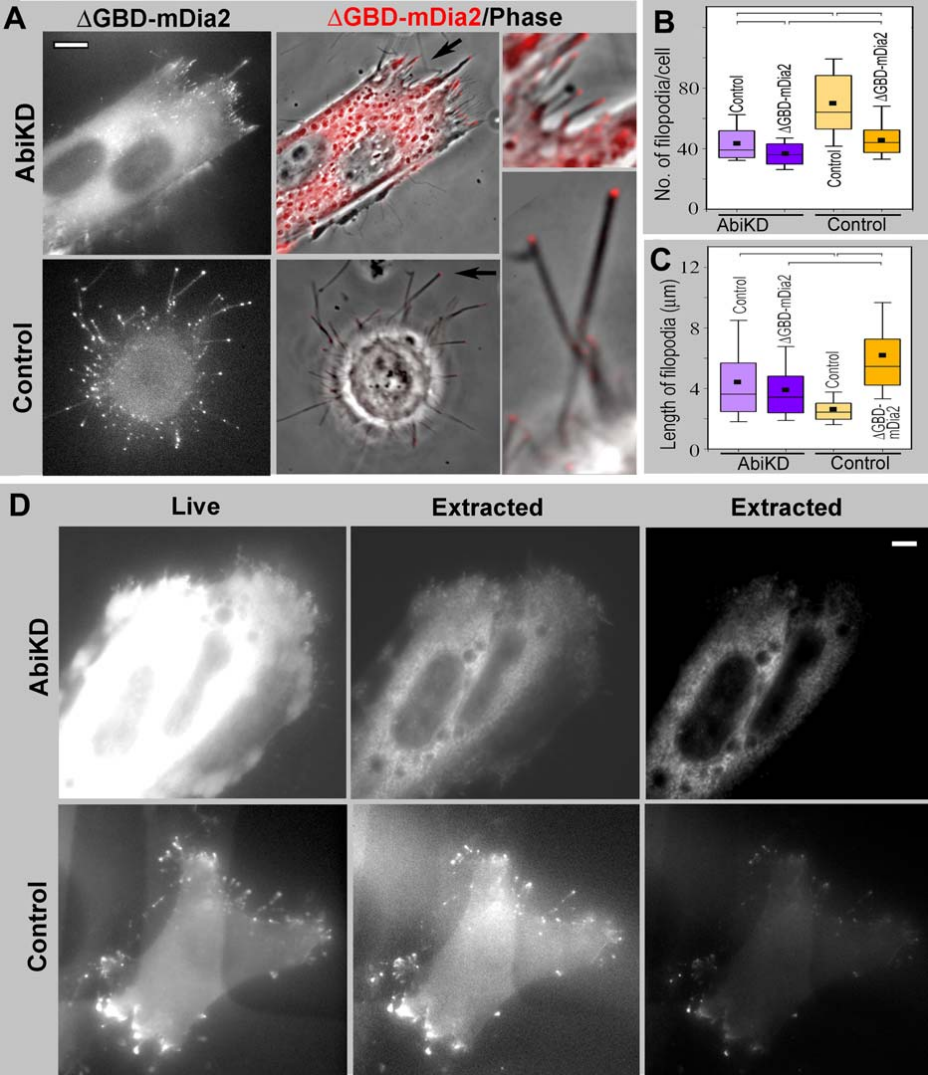
Phenotype of ΔGBD-mDia2 Expression in Abi1KD Cells (A) GFP-ΔGBD-mDia2 in Abi1KD cells (top row) is not efficiently targeted to the membrane and weakly induces filopodia, as compared to control HeLa cells (bottom row). GFP is shown in the left column and phase contrast overlaid with GFP in red in the middle column. Filopodia indicated by arrows are identically enlarged (approximately 3-fold) in insets at right. (B and C) Filopodia number (B) and length (C) in Abi1KD and control HeLa cells after transfection with ΔGBD-mDia2. Box-and-whisker plots are as in Figure 1. Differences between datasets connected by brackets are statistically significant (*p* < 0.001, *n* = 265–1,102 filopodia from 11–31 cells). (D) GFP-ΔGBD-mDia2 in Abi1KD cells is more sensitive to detergent extraction than in control HeLa cells. Abi1KD (top) or control (bottom) cells expressing GFP-ΔGBD-mDia2 are shown live (left) or detergent-extracted (middle and right) after identical image processing (middle) or contrast-enhanced to better visualize tips of filopodia (right). Scale bars indicate 10 μm.

Similar to B16F1 cells, expression of ΔGBD-mDia2 in HeLa cells induced long, robust filopodia (Figure 12A and 12C). Interestingly, the number of filopodia decreased after ΔGBD-mDia2 expression (Figure 11B), which might occur because of extensive fusion of neighboring filopodia, a well-known feature of filopodia dynamics [40]. In contrast, Abi1KD cells formed slender and significantly shorter filopodia in the same conditions (Figure 11A and 11C), whereas their number slightly, but significantly, decreased compared to ΔGBD-mDia2–expressing control cells (Figure 12B). Furthermore, ΔGBD-mDia2 in Abi1KD cells localized cytoplasmically, with only faint signal at the filopodial tips, which was more sensitive to detergent extraction than in control HeLa cells (Figure 12D). Thus, the ΔGBD-mDia2 in Abi1KD cells, similar to FH1FH2-mDia2 in B16F1 cells, was deficient in robust targeting to the edge and efficient induction of filopodia, suggesting a role of Abi1 in ΔGBD-mDia2 targeting.

## Discussion

In this study, by combination of loss-of-function (RNAi) and gain-of-function (constitutively active mutant) approaches, we investigated a role of mDia2 formin in the actin-based protrusion in motile cells. Although our original goal was to characterize the mechanism of mDia2-dependent filopodia formation, we unexpectedly found that mDia2 is important for lamellipodial protrusion and induces filopodia in association with lamellipodia. Based on our data, we propose a model suggesting that mDia2 is recruited to the lamellipodial leading edge in an Abi1-dependent manner, where it nucleates new filaments and/or maintains filament elongation by protecting barbed ends from capping. In the course of elongation, lamellipodial filaments converge and become bundled, generating filopodia.

### Targeting of mDia2 to the Leading Edge

We found that mDia2 can be recruited to the leading edge by at least two mechanisms, one of which relies on binding of the mDia2 FH1FH2 module to barbed ends and another on N-terminal mDia2 sequences interacting with Abi1. The FH1FH2 module contributes to localization to filopodial tips by binding to and riding on elongating barbed ends, which accumulate at the membrane after encountering the boundary conditions [25]. However, FH1FH2-mDia2 was not sufficient to fully restore formin's functions in mDia2-interferred cells and to efficiently induce filopodia, likely reflecting inferior membrane targeting of FH1FH2-mDia2 as compared to ΔGBD-mDia2 or full-length mDia2. In the second mechanism, binding of N-terminal regulatory sequences of mDia2 to Abi1 provides a potential additional surface for mDia2 proper localization to leading edges. This is also consistent with the observation that similar regions were previously shown to control targeting of other formins [41,42]. It is also possible that Abi1 not only recruits mDia2 to the membrane, but also participates in its regulation, but we currently do not have data to evaluate this idea.

The majority of Abi1 has been shown to be engaged in complex with WAVE, Nap1, and PIR121 [11,45,48]. However, we did not detect interaction of mDia2 with WAVE, Nap1, or PIR121, indicating that mDia2 and Abi1 may form a distinct macromolecular signaling unit with respect to the WANP complex. Accordingly, only a minor fraction of Abi1 interacted with mDia2 in coimmunoprecipitation experiments. The ability of Abi1 to act as a scaffolding molecule driving the formation of additional signaling units, such as those containing N-WASP [14] or Eps8 [49,50], has been previously observed. The Abi1-mDia2 assembly adds to the versatility of Abi1, which by entering distinct complexes is capable of controlling diverse processes and/or coordinating related activities, such as the formation of lamellipodia and filopodia protrusions reported here. The precise regulatory events governing the relationship between these units remain to be defined and represent one of the challenges for future investigation.

### Lamellipodia Protrusion

Specific targeting of proteins is a common way to confine the protein activity to a certain area. Therefore, mDia2 targeting to lamellipodia and filopodia points to an idea that mDia2 may function there. Although mDia2′s role in filopodia is expected [16,17], the lamellipodial function contrasts with the current belief that formins and Arp2/3 complex are responsible for different actin structures. Two complementary sets of data in our study suggest that mDia2 is an important player in lamellipodial protrusion. First, mDia2 inhibition by siRNA severely impaired lamellipodia and reduced long filaments in the remaining lamellipodial network. Second, constitutively active ΔGBD-mDia2, conversely, induced numerous long filaments in lamellipodia. Similar complementarity was observed regarding the persistence of lamellipodia protrusion, which decreased after mDia2 depletion and increased after expression of the constitutively active mutant. The lamellipodial function seems specific for mDia2; other formins, which were supposedly present in knockdown cells, were not sufficient. Moreover, overexpression of a related formin, mDia1, did not rescue lamellipodia formation. Interestingly, 3T3 cells lacking mDia2 [15] still make lamellipodia. We found, however, that 3T3 cells express another related formin, mDia3, which is absent in B16F1 cells (unpublished data), raising a possibility that mDia3 may be partially redundant with mDia2.

**Figure 13:**
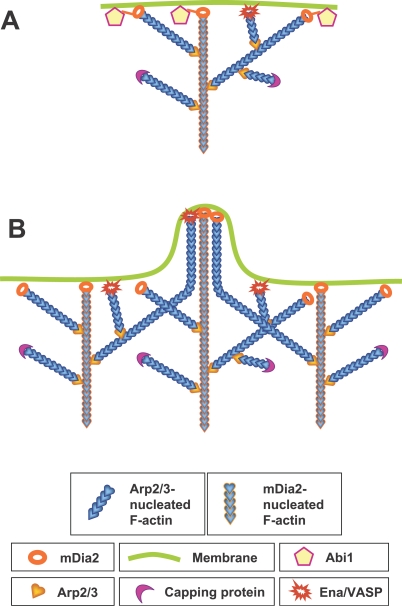
Models for mDia2 Functions in Lamellipodia and Filopodia (A) In lamellipodia, mDia2 is targeted to the membrane in an Abi1-dependent manner, where it may nucleate “mother” filaments, which serve as a base for Arp2/3-dependent nucleation, and/or protect from capping elongating barbed ends, which are nucleated by mDia2 itself or by Arp2/3 complex. Other barbed ends may be protected from capping by VASP. Unnecessary barbed ends are capped by capping protein. (B) During formation of filopodia, lamellipodial filaments associated at their barbed ends with mDia2 or VASP converge during elongation and become cross-linked, thus forming filopodial bundles.

Biochemical activities of mDia2 suggest that it may contribute to lamellipodia formation through nucleation and/or anticapping protection of actin filaments (Figure 13A). Our ΔGBD-mDia2 data are consistent with both possibilities. Thus, high frequency of linear filaments with unlinked “pointed” ends, and fast depolymerization of actin filaments from the rear (see Figure 8) are consistent with formin, but not with Arp2/3-mediated nucleation. The anticapping activity of mDia2 is also employed during lamellipodia formation. Indeed, the dissociation of mDia2 from barbed ends is very slow, at least in vitro [51], suggesting that it would protect the nucleated filaments from capping for long time. In addition to this consideration, we found that (1) capping protein was diminished in ΔGBD-mDia2–rich lamellipodial regions; (2) ΔGBD-mDia2 enrichment at the leading edge correlated with the presence of long actin filaments in EM images; and (3) mDia2-depleted lamellipodia contained very short branched filaments. The latter observation also suggests that mDia2 may protect from capping branched filaments, apparently nucleated by Arp2/3 complex. The ability of ΔGBD-mDia2 to displace VASP from barbed ends (Figures 6A and S6) is also consistent with this idea. Notably, long unbranched filaments may also form in the course of debranching of Arp2/3-nucleated and mDia2-protected filaments without nucleation by ΔGBD-mDia2. Importantly, lamellipodial filaments nucleated and/or protected from capping by mDia2 may be responsible for the presence of long filaments [52], and two kinetically distinct actin subpopulations in lamellipodia [53] noted earlier.

It is unclear why mDia2 has such a significant role in lamellipodial protrusion, considering the well-established involvement of Arp2/3 complex in this process [1]. One possibility is that mDia2-nucleated filaments may help to initiate lamellipodia by nucleating “mother” filaments, which are required to Arp2/3-dependent nucleation. Additionally, mDia2 may be a better “device,” compared to VASP, for example, to maintain fast and processive barbed-end growth and thus to ensure persistent protrusion. Long filaments induced by mDia2 in lamellipodia may also provide better connection of lamellipodia to the rest of the cytoskeleton. In any case, our results suggest that two different actin nucleators, Arp2/3 complex and mDia2, jointly contribute to generation of lamellipodia.

### Filopodia Protrusion

A role of mDia2 in filopodia formation in mammalian cells [16,17] and of dDia2 in *Dictyostelium* was reported [18], but the mechanism of formin-mediated filopodia induction has not been fully investigated. The previous model suggested that mDia2 initiates filopodia from a focal spot not necessarily associated with lamellipodia [17,22], thus putting this mechanism at an apparent conflict with the convergent elongation model [19]. In this study, we provide evidence to solve this contradiction. By investigating the spatiokinetic mechanism underlying filopodia initiation by active mDia2, we found that, in our experimental system, lamellipodia are a highly preferred site for filopodia initiation, although subsequently, filopodia may lose association with lamellipodia, for example, by moving to the dorsal surface. However, we cannot exclude a possibility that in other experimental systems or conditions [12,54], formins may nucleate actin bundles from focal spots without association with lamellipodia or other dendritic arrays.

The overall process of filopodia initiation by mDia2 was very similar to that described for naturally emerging filopodia [19] and involved gradual convergence of lamellipodial filaments (Figure 13B). These results show that the convergent elongation mechanism is applicable to formin-induced filaments. Importantly, FL-mDia2 displayed similar convergence of lamellipodial fluorescence into dots, as ΔGBD-mDia2, suggesting that convergence behavior is not entirely due to excessive activity of ΔGBD-mDia2. Initiation of filopodia by ΔGBD-mDia2 was very efficient, whereas the lamellipodia from which the filopodia arose were quite transient. This can be explained by the favorable combination of biochemical activities in mDia2, which may drive the efficient transformation of lamellipodia into filopodia, not only by nucleating and elongating filaments, but also by cross-linking them [55] and thus promoting the initiation of filament convergence. A downside of the excessive activity of ΔGBD-mDia2 is that filopodia acquired an abnormal club-like shape, in which the majority of filaments did not extend all the way to the cell body. Based on the known mechanism of formin-mediated nucleation and our data obtained by correlative light microscopy and EM (Figure 8), and by observing GFP-actin dynamics (Figure 6), we interpret this phenotype as fast depolymerization of mDia2-nucleated filaments from unprotected pointed ends.

Because of formation of atypical club-like filopodia, we suppose that ΔGBD-mDia2 does not fully recapitulate the physiological pathway of filopodia formation. Based on the structural data, we previously proposed that Arp2/3 complex nucleates filaments for filopodial bundles [19]. We have confirmed this idea by a functional siRNA approach showing that Arp2/3 depletion inhibits filopodia (F. Korobova and T. Svitkina, unpublished data. These two sets of data together suggest that Arp2/3 complex and mDia2 may jointly contribute to filopodia formation. In such case, one would expect that the balanced activities of these two nucleators are important for the normal filopodial shape and functions, whereas any imbalance will likely lead to aberrant filopodia. Club-like filopodia induced by constitutive activation of mDia2 may serve as an illustration for this contention. Abnormal filopodia were also formed after depletion of mDia2. However, the relative roles of formins and Arp2/3 complex in filopodia await further investigation.

In summary, we have shown that mDia2 plays a role in formation of both lamellipodia and filopodia. However, these roles are not separate, but related to each other; mDia2 first participates in lamellipodia formation and then induces filopodia from lamellipodia. A most likely scenario is that mDia2-nucleated actin filaments and/or filaments nucleated by Arp2/3 complex and protected by mDia2 are initially dispersed in the lamellipodial network, and subsequently, they elongate persistently and gradually converge, eventually segregating themselves into filopodial bundles. Although regulation of actin dynamics is the most straightforward way to explain how mDia2 may function in protrusion, we cannot exclude a possibility that other mDia2 activities, such as regulation of microtubule dynamics [56], membrane trafficking [30,31], surface blebbing [57], or transcription [32], also contribute to generation of observed phenotypes.

## Materials and Methods

### Reagents.

*Plasmids.* Full length mDia2 in a Bluescript cloning vector (Stratagene) and pEFm-EGFP-ΔGBD-mDia2 (amino acids [aa] 258–1,171) [15] were gifts of A. Alberts (Van Andel Research Institute). The mRFP1-ΔGBD-mDia2 construct was prepared by substituting EGFP with mRFP1 [58], which is a gift from R. Tsien (University of California San Diego). Full-length mDia2 was cloned into pEGFP-C1 vector (Clontech). DNA fragments for FH1 (aa 519–600), trFH1FH2 (aa 519–909), FH1FH2 (aa 519-1007), and NT-mDia2 (aa 1–550) were amplified by PCR, cloned into pEGFP-C1 vector, and confirmed by sequencing. EGFP-VASP was obtained from F. Gertler (Massachusetts Institute of Technology [MIT]) and cloned into pECFP-C1 vector (Clontech); myosin X [36] from R. Cheney (University of North Carolina-Chapel Hill); and full length mDia1 and ΔN2-mDia1 [44] from S. Narumiya (Kyoto University).

*Antibodies.* The following polyclonal antibodies were obtained as gifts: mDia2 from A. Alberts [15] and H. Higgs (Dartmouth Medical School); CPβ2 C terminus (R26) from D. Schafer (University of Virginia); Arp3 from T. Uruno (Holland Laboratory); cofilin from J. Condeelis (Albert Einstein College of Medicine); and VASP from F. Gertler (MIT). Antibody against Abi1 was previously described [59]. The following antibodies were from commercial sources: fascin (DAKO), α-tubulin (Sigma), cortactin 411F (Upstate), and secondary antibodies (Molecular Probes or Jackson Laboratories). All other reagents were from Sigma unless indicated otherwise.

*siRNA*. siRNA for mDia2 coding region (5′-ataagagagcagtatttcaaa-3′) and control siRNAs (5′-aagaaatagggaaggtggaac-3′ and 5′-aaatttacaggacttcagtca-3′) were obtained from Dharmacon, and siRNA for DIAPH3 coding region (5′-aaccttcggatttaaccttag-3′) was from Ambion. siRNAs were Cy3-labeled using a Silencer siRNA labeling kit (Ambion) and used at 20 nM concentration. The efficiency of siRNA transfection was approximately 90%. Effects of siRNA were analyzed 2 to 3 d post-transfection. For rescue experiments, silent mutations were introduced into siRNA-targeted region of GFP-mDia2 (5′-ataagagaAcagtaCttcaaa-3′) using the Quikchange site-directed mutagenesis kit II (Stratagene).

### Cell culture and microscopy.

B16F1 mouse melanoma cells [19], HeLa cells, and Abi1KD HeLa cells [11] were cultured as described. Transient transfection of DNA and siRNA was performed using Fugene6 or Lipofectamine 2000 (Invitrogen), respectively. Lipofectamine 2000 was used for cotransfection of siRNA and DNA. Light microscopy was performed using Eclipse TE2000-U inverted microscope (Nikon) equipped with Planapo 100 × 1.3 NA or 20 × 0.75 objectives and Cascade 512B CCD camera (Photometrics) driven by Metamorph imaging software (Molecular Devices). For live-cell imaging, cells were transferred into phenol red–free L-15 or DMEM medium (Gibco) supplemented with 10% FBS and kept on the microscope stage at 35 °C during observation.

Samples for platinum replica EM were processed as described [60] and analyzed using JEOL 1200EX transmission electron microscope operated at 120 kV. Cells expressing ΔGBD-mDia2 for EM analysis were identified by correlative EM or isolated by FACS. Immunostaining was performed after cell extraction for 5 min at room temperature with 1% Triton X-100 in PEM buffer (100 mM PIPES-KOH [pH 6.9] 1 mM MgCl_2_, 1 mM EGTA) containing 2% polyetheleneglycol (MW 35,000) and 2 μM phalloidin, followed by fixation with 0.2% glutaraldehyde and quenching with NaBH_4_. Texas red phalloidin (0.033 μM; Molecular Probes) was used for actin staining. Fascin staining was performed after methanol fixation of extracted cells. For staining with VASP or mDia2 antibody, cells were extracted/fixed by a mixture of 0.25% glutaraldehyde and 0.5% Triton X-100 in PEM buffer for 20 min. To evaluate cytoskeletal association of mDia2 constructs, cells were first imaged live, then culture medium was replaced by the extraction solution, the same as used for immunostaining, and another image was acquired approximately 2 min later.

### Image analysis and statistics.

All morphometric measurements were done using MetaMorph Imaging software (Molecular Devices) unless stated otherwise. Graphs and statistical analysis were done using SigmaPlot software. Statistical significance was determined by the Student *t*-test.

Quantification of lamellipodia and filopodia were performed as described [20]. Briefly, MetaMorph line function was used to trace and measure the whole-cell perimeter and the cell perimeter with adjacent lamellipodia (an actin-rich fringe with fluorescence intensity gradually declining with the distance from the edge) on fixed phalloidin-stained cells. The fraction of the cell perimeter occupied by lamellipodia was used as a parameter for quantification. For quantification purposes, filopodia were defined as actin-rich finger-like protrusions crossing the cell edge and having fluorescence intensity at least 1.2-fold above background. Quantification was performed using a blind experimental procedure by an uninformed observer on coded samples. Control and experimentally treated cells in mixed populations were measured using phalloidin channel only. After numbers were assigned to individual cells, the phalloidin images were combined with other channels showing siRNA or a rescue construct, the identity of samples was decoded, and numbers were entered into respective columns of a spread sheet for statistical analysis.

For spreading assays, the projected cell area was measured 2 h after plating. For cell migration analysis, cells transfected with mDia2 siRNA (Cy3 labeled) or control siRNA (nonlabeled) were co-cultured in 35-mm dishes and imaged 36–48 h post-transfection (4 h after plating). Phase contrast time-lapse sequences were acquired with 5-min intervals for 6 h using a 4× objective. Fluorescence images to identify siRNA-transfected cells were acquired immediately before and after each movie. Cell positions were recorded every ten frames using Track Object tool in MetaMorph. An average instantaneous rate was calculated for each cell, and then the mean speed of cell migration was determined for each group of cells. To analyze the lamellipodial dynamics, time-lapse sequences were acquired with 3-s intervals for 10 min using a 100× objective. Kymographs were generated along straight lines drawn in the direction of protrusion. Rates of lamellipodia protrusion were determined based on slopes produced by advancing leading edges. Persistence corresponds to time intervals during which individual protrusions occurred. The relative mDia2 levels in control and mDia2 siRNA-treated cells were determined by immunostaining after fixation with paraformaldehyde and permeabilization with Triton X-100. Integrated fluorescence intensity of mDia2 staining for each cell after background subtraction was plotted against the fraction of the cell edge occupied by lamellipodia.

To determine the degree of mutual orientation of actin filaments in lamellipodia of control and ΔGBD-mDia2-expressing cells, square EM images of the lamellipodial network ranging from 0.13 to 0.53 μm^2^ were thresholded using Adobe Photoshop to maximally highlight the linear features. Matlab software was used to generate two-dimensional Fast Fourier Transform from these images, collect radial intensity line scans, plot intensity as a function of angle, and fit a Gaussian curve to the major peak. Standard deviation of the Gaussian fit was used as a parameter of the orientational order. Matlab code for this analysis was provided by J. Winer, Q. Wen, and P. Janmey (University of Pennsylvania).

### Biochemical assays.

For immunoblotting, cells were lysed in buffer containing 10 mM Tris (pH 7.5), 150 mM NaCl, 1% Triton X-100, 10% glycerol, and a protease inhibitor tablet (Roche). Protein concentration of the lysates was determined using Bio-Rad protein assay kit (Bio-Rad). Proteins were separated by SDS-PAGE (7.5%–10% polyacrylamide). Tubulin was used as loading control. Immunoblots were developed using ECF Western blotting kit (Amersham).

Coimmunoprecipitation and in vitro binding assays were performed as described [59]. For coimmunoprecipitation, total cellular lysates (1 mg) of 293T cells cotransfected with GFP-FL-mDia2 and Abi1, alone or in combination, were immunoprecipitated with an Abi1 antibody. For GST pull-down assay, total cellular lysates (1 mg) of 293T cells transfected with GFP-mDia2 were incubated with 1.5 μM of immobilized GST-Abi1, GST-Abi1-SH3 (aa 330–480), or GST, as a control. Lysates (20 μg) and bound proteins in both cases were resolved by SDS-PAGE and immunoblotted. GFP antibody was used to detect GFP-mDia2 constructs.

## Supporting Information

Figure S1 The Correlation of the Intensity of mDia2 Immunostaining with Lamellipodia ExpressionEach data point represents an individual cell.a.u., arbitrary units. (183 KB PDF)

Figure S2 mDia2 Knockdown and Its Rescue by FL-mDia2*, but Not by Rac1V12 Cell populations transfected with mDia2 siRNA (A) or cotransfected with mDia2 siRNA and siRNA-resistant GFP-FL-mDia2* or GFP-RacV12 (B). mDia2 knockdown inhibits lamellipodia; this phenotype can be rescued by FL-mDia2*, but not by GFP-Rac1V12. Bars indicate 25 μm.
(9.3 MB PDF)

Figure S3 Immunostaining of mDia2 and Abi1 Distribution of endogenous mDia2 (red) and Abi1 (green) in lamellipodia of B16F1 cell, as detected by immunostaining. Bar indicates 5 μm.
(1.9 MB PDF)

Figure S4 The 3D Structure of ΔGBD-mDia2–Induced Lamellipodia Both unbound and branched proximal (“pointed”) ends of actin filaments can be detected in ΔGBD-mDia2–induced lamellipodia.
Top: anaglyph stereo image (right eye blue) showing 3D organization of filaments in ΔGBD-mDia2–induced lamellipodia.
Bottom: 2D image of the same region with unbound ends marked by yellow dots, and ends engaged in branch formation by red dots. Boxed region is enlarged in the inset with branched filaments highlighted in blue. Scale bar indicates 0.2 μm.
(10.3 MB PDF)

Figure S5 Dynamics of Filopodia Formation in B16F1 Cells Expressing ΔGBD-mDia2 Examples show formation of a club-like filopodium (arrowhead) and a dorsal filopodium (arrow).
Top: phase contrast.
Middle: GFP fluorescence in inverse contrast.
Bottom: overlay with GFP in red.
Arrowhead points to the formation of a club-like filopodium by fusion of several smaller filopodia. Discontinuous linear fluorescence of ΔGBD-mDia2 (0:00 time point) gradually converges (0:30) and produces two distinct dots at the tips of small filopodia (1:00). Another filopodium is seen in-between with barely detectable GFP signal. The right filopodium moves laterally (1:00 through 2:00), and all three filopodia fuse (2:30), producing a single filopodium that protrudes extensively and acquires a club-like shape.
Arrow points to the formation of a dorsal protrusion from a lateral filopodium. Linear fluorescence at the lower right side of the lamellipodium (0:30) gradually produces two filopodia by the 2:00 time point, which fuse (3:00), and the resulting structure translocates to the dorsal surface of lamella.
(6.9 MB PDF)

Figure S6 Phenotypes Induced by Expression of ΔN2-mDia1 or Inactive mDia2 Mutants in B16F1 Cells and by ΔGBD-mDia2 in Ena/VASP-Deficient Cells (A) Phenotype induced by expression of GFP-ΔN2-mDia1. GFP fluorescence of GFP-ΔN2-mDia1 (left) and F-actin enrichment (middle) are found throughout the cytoplasm of a bipolar cell as previously described in other cells [43]. Only very short, finger-like protrusions with slight enrichment of ΔN2-mDia1 at the tips could be observed at the cell edges abutted by actin bundles (right). No filopodial-like protrusions reaching significant length were observed. Arrow in the merged panel points to a region enlarged at right.
(B) Expression of GFP-tagged mDia2 constructs in B16F1 cells. FH1 domain, residues 519–600 (FH1), and truncated FH1FH2, residues 519–909 (trFH1FH2) have cytoplasmic distribution and do not induce filopodia. Scale bars indicate 10 μm.
(C) Filopodia induction does not depend on Ena/VASP proteins. Expression of GFP-ΔGBD-mDia2 (a and b) or mRFP1-ΔGBD-mDia2 (c) in Ena/VASP-deficient MV^D7^ cells (a) or MV^D7^ cells stably re-expressing GFP-Mena (MVD7-EM) (b) or transiently re-expressing GFP-VASP (c). (a and b) ΔGBD-mDia2 localizes to the membrane and induces filopodia equally well in MV^D7^ and MV^D7^-EM cells. (c) In cells expressing relatively low levels of ΔGBD-mDia2 (top row), re-expressed VASP is still occasionally present at filopodial tips (arrow), but is displaced to more proximal regions of filopodia (arrowheads) in highly expressing cells (bottom row). Scale bars indicate 5 μm in (a and b) and 10 μm in (c).
(4 MB PDF)

Figure S7 Characterization of Abi1KD HeLa Cells (A) Western blotting of lysates from control or Abi1KD HeLa cells. Amount of protein loaded is shown in μg. Expression of Abi1 is decreased by approximately 90% in Abi1KD cells, but Arp2/3 subunit p34-Arc (p34) is not changed.
(B) EM of a peripheral region of a control HeLa cell.
(C) EM of a peripheral region of Abi1KD cell with two filopodia. Boxed regions showing branched filaments in filopodial roots are enlarged at the bottom as 3D anaglyph images (right eye red) (top row), and as 2D images with branched filaments highlighted in color (bottom row). Although lamellipodia are grossly inhibited in these cells, small regions of dendritic network can be occasionally detected at cell edges (arrow).
Bars indicate 0.5 μm.
(8.8 MB PDF)

Video S1 Inhibition of Protrusive Activity of B16F1 Cell Transfected with mDia2 siRNA B16F1 cell at the left is transfected with mDia2 siRNA and has very low protrusive activity. Control untransfected B16F1 cell at the right forms lamellipodia and filopodia. This video corresponds to Figure 1F.
(1.2 MB MOV)

Video S2 Another Example of B16F1 Cell Transfected with mDia2 siRNA Lamellipodia and filopodia are severely inhibited.
(19.4 MB MOV)

Video S3 Formation of Dynamic Filopodia by GFP-ΔGBD-mDia2 Expression of GFP-ΔGBD-mDia2 (red) induces formation of dynamic filopodia in a B16F1 cell. GFP-ΔGBD-mDia2 remains associated with filopodial tips during protrusion. This video corresponds to Figure 6C.
(1.4 MB MOV)

Video S4 Kinetics of GFP-Actin during Filopodia Formation Induced by mRFP-ΔGBD-mDia2 in B16F1 Cell Coexpression of GFP-actin (white) and mRFP1-ΔGBD-mDia2 (not shown) in B16F1 cell shows actin enrichment at the thicker termini of GFP-ΔGBD-mDia2–induced filopodia. Arrow points to an actin speckle which undergoes slow retrograde flow and finally disappears, while the filopodial tip protrudes forward. This behavior illustrates actin assembly at the tip, whereas disappearance of the speckles and overall decrease of actin intensity toward the rear indicates actin disassembly in proximal regions. This video corresponds to Figure 6F–6H.
(5.9 MB MOV)

Video S5 Kinetics of GFP-ΔGBD-mDia2 during Filopodia Formation in B16F1 Cells Linear fluorescence along the lamellipodial leading edge gradually transforms into a series of bright dots at the filopodial tips. This video corresponds to Figure 8A.
(886 KB MOV)

Video S6 Kinetics of GFP-FL-mDia2 during Filopodia Formation in B16F1 Cells Linear fluorescence along the lamellipodial leading edge converges into dots at the filopodial tips (arrows).
(2 MB MOV)

Video S7 GFP-ΔGBD-mDia2 Expressing B16F1 Cell Used for Correlative EM with Known History Top: fluorescence; bottom: phase overlaid with fluorescence in red. This video corresponds to Figure 8B.
(1.4 MB MOV)

Video S8 Formation of a Nascent Filopodium (∼30 second old) in GFP-ΔGBD-mDia2–Expressing B16F1 Cell Top: fluorescence; bottom: phase overlaid with fluorescence in red. This video corresponds to Figure 8D and 8E.
(328 KB MOV)

Video S9 Formation of a Nascent Filopodium (∼90 second old) in GFP-ΔGBD-mDia2–Expressing B16F1 Cell Top: fluorescence; bottom: phase overlaid with fluorescence in red. This video corresponds to Figure 8F and 8G.
(626 KB MOV)

Video S10 Kinetics of GFP-FH1FH2-mDia 2 in B16F1 Cell The time-lapse sequence of B16F1 cell expressing GFP-FH1FH2-mDia2. GFP-FH1FH2-mDia2 displays strong cytoplasmic localization and is mildly enriched at the tips of dynamic filopodia.
(1 MB MOV)

## Abbreviations

Abi1KD: Abi1-RNAi-knockdown cells
GFP: green fluorescent protein
RNAi: RNA interference
siRNA: short interfering RNA
WANP complex: WAVE/Abi1/Nap1/PIR121/HSP300 complex
