# Supplementary figures and images for: Novel Roles of Formin mDia2 in Lamellipodia and Filopodia Formation in Motile Cells
Source: PLoS Biol. 2007 Nov 27;5(11):e317. doi: 10.1371/journal.pbio.0050317 (PMC2229861; doi:10.1371/journal.pbio.0050317)

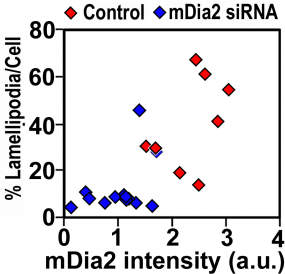

Supplement: Figure S1 — (183 KB PDF) [file pbio.0050317.sg001.pdf]

F-actin

A

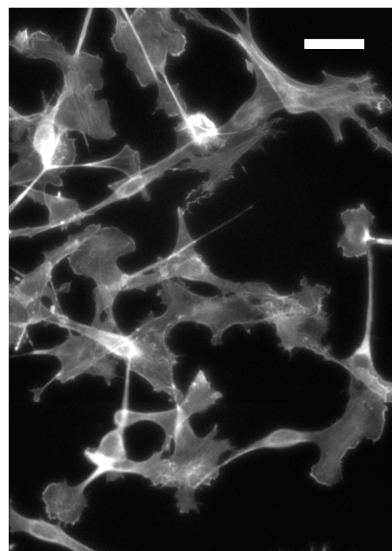

mDia2 siRNA

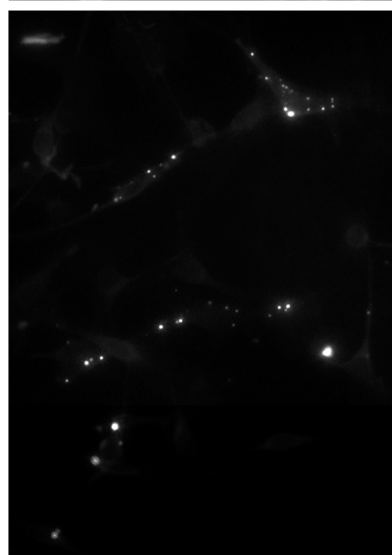

Merge

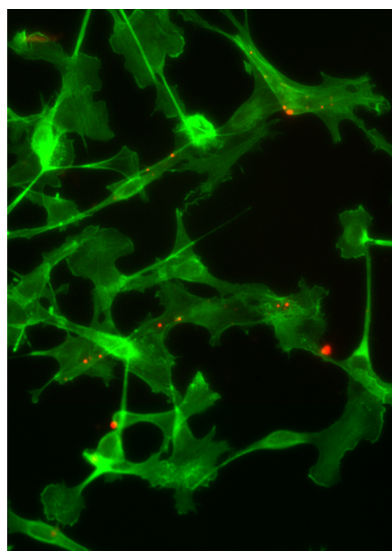

GFP

B

GFP-mDia2\*

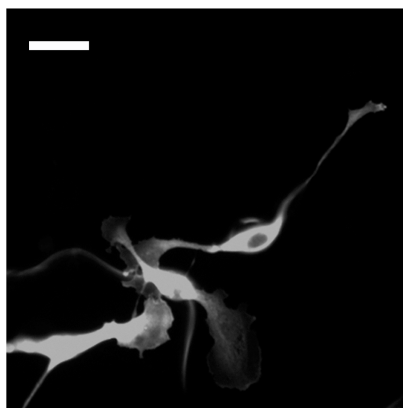

mDia2 siRNA

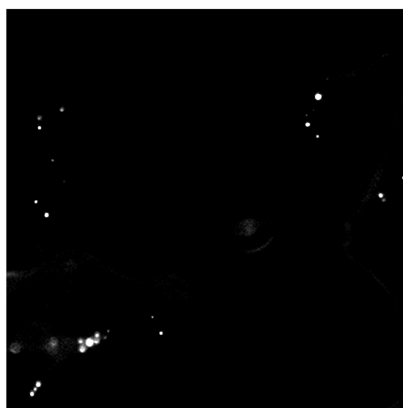

F-actin

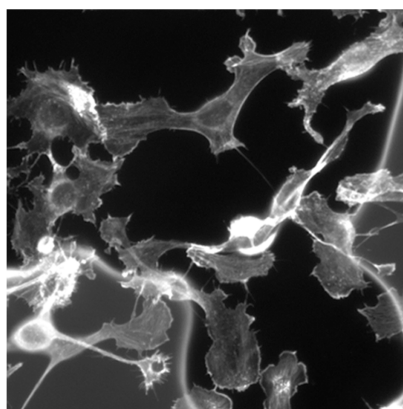

Merge

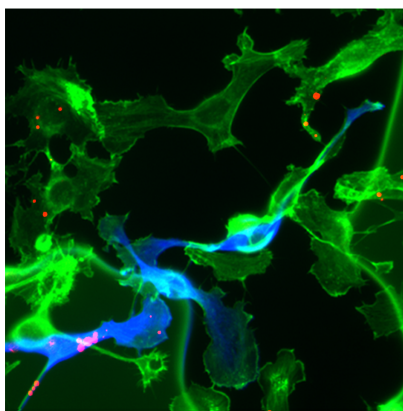

GFP-V12RAC1

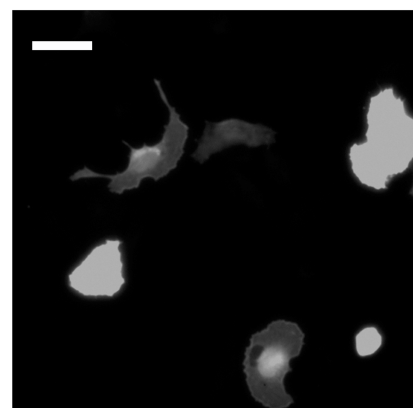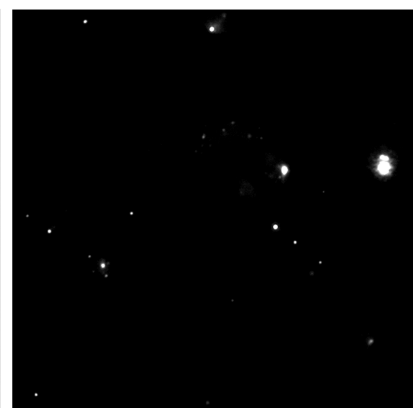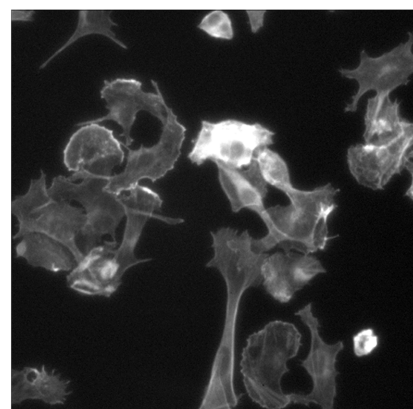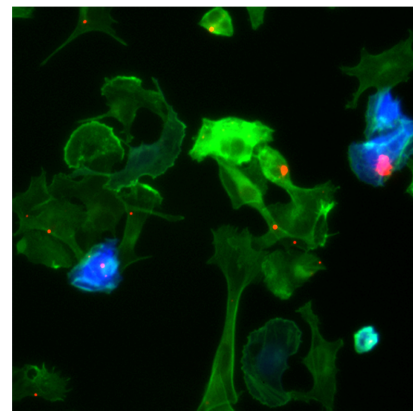

Supplement: Figure S2 — Cell populations transfected with mDia2 siRNA (A) or cotransfected with mDia2 siRNA and siRNA-resistant GFP-FL-mDia2* or GFP-RacV12 (B). mDia2 knockdown inhibits lamellipodia; this phenotype can be rescued by FL-mDia2*, but not by GFP-Rac1V12. Bars indicate 25 μm. (9.3 MB PDF) [file pbio.0050317.sg002.pdf]

**mDia2**

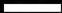

**Abi1**

**Merge**

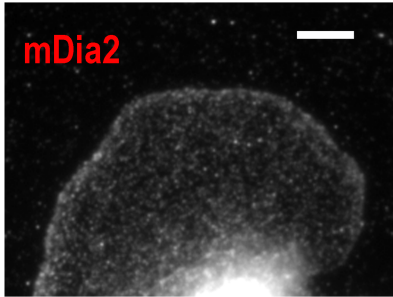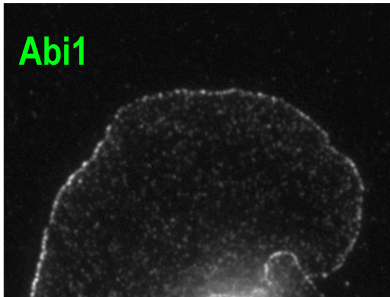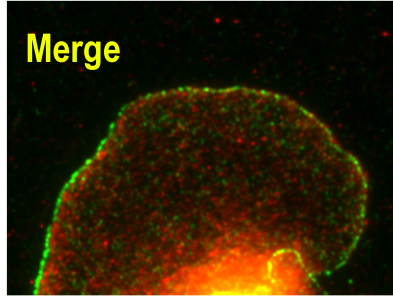

Supplement: Figure S3 — Distribution of endogenous mDia2 (red) and Abi1 (green) in lamellipodia of B16F1 cell, as detected by immunostaining. Bar indicates 5 μm. (1.9 MB PDF) [file pbio.0050317.sg003.pdf]

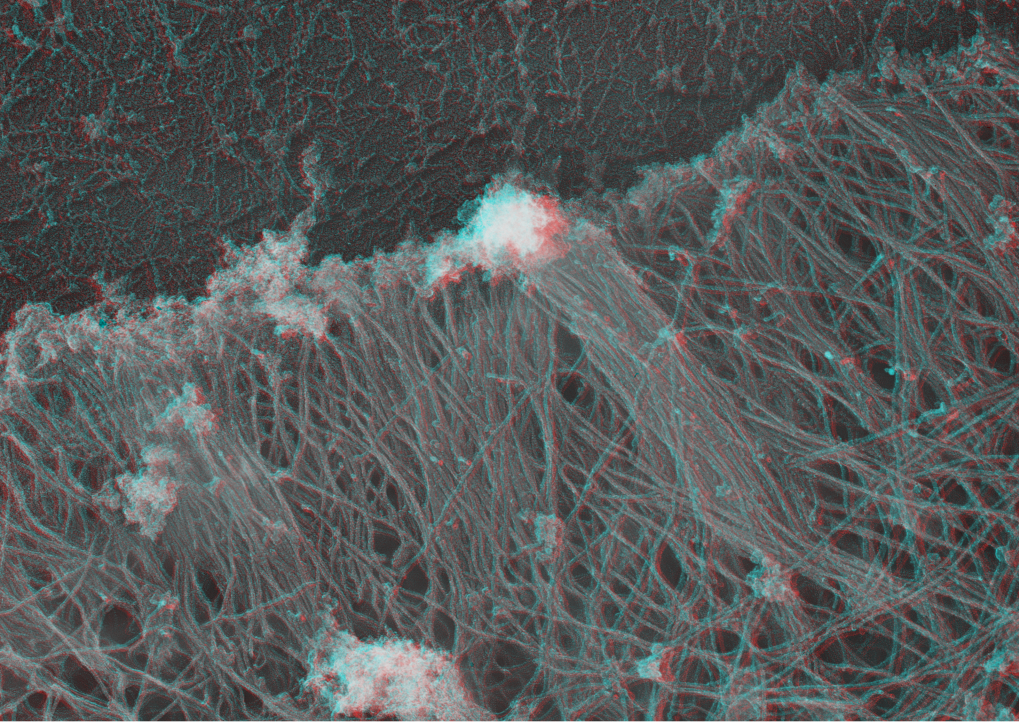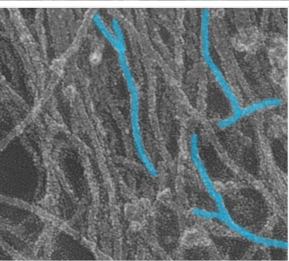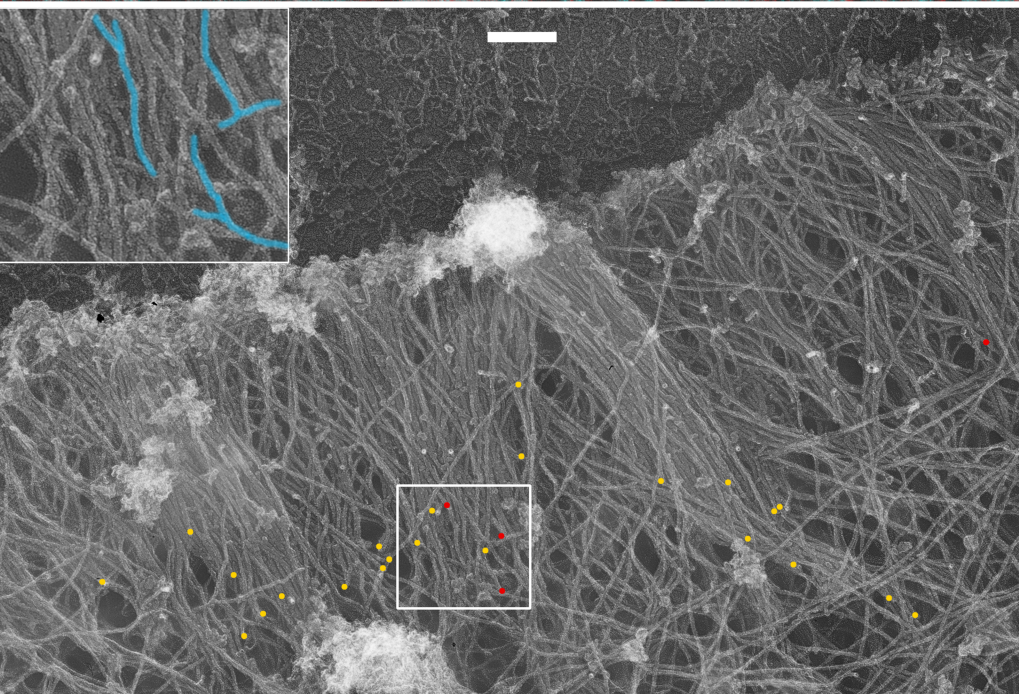

Supplement: Figure S4 — Both unbound and branched proximal (“pointed”) ends of actin filaments can be detected in ΔGBD-mDia2–induced lamellipodia. Top: anaglyph stereo image (right eye blue) showing 3D organization of filaments in ΔGBD-mDia2–induced lamellipodia. Bottom: 2D image of the same region with unbound ends marked by yellow dots, and ends engaged in branch formation by red dots. Boxed region is enlarged in the inset with branched filaments highlighted in blue. Scale bar indicates 0.2 μm. (10.3 MB PDF) [file pbio.0050317.sg004.pdf]

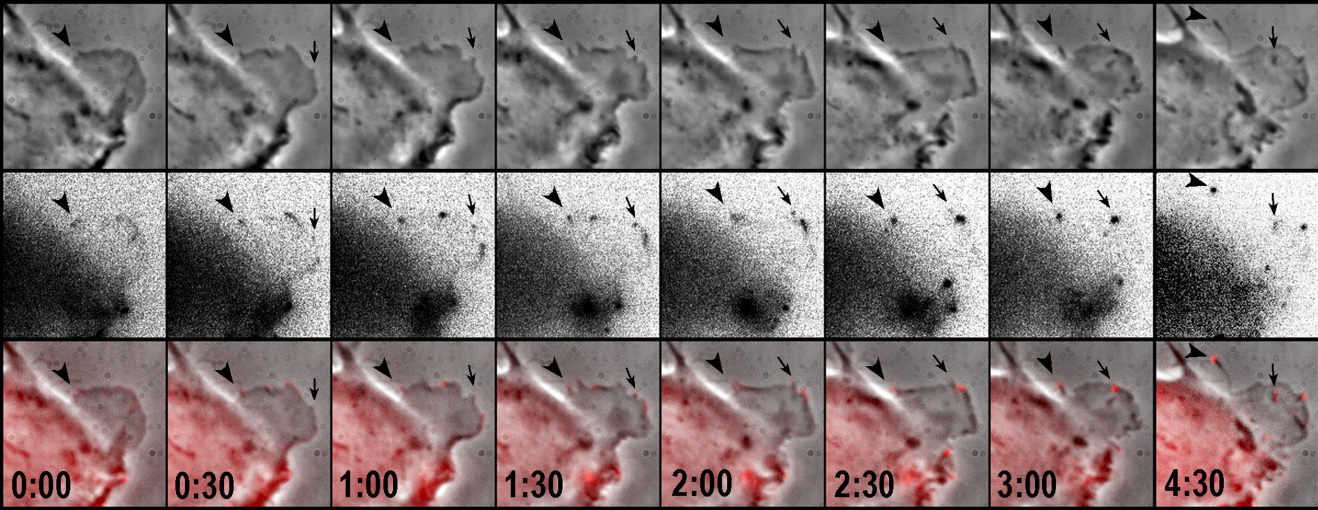

Supplement: Figure S5 — Examples show formation of a club-like filopodium (arrowhead) and a dorsal filopodium (arrow). Top: phase contrast. Middle: GFP fluorescence in inverse contrast. Bottom: overlay with GFP in red. Arrowhead points to the formation of a club-like filopodium by fusion of several smaller filopodia. Discontinuous linear fluorescence of ΔGBD-mDia2 (0:00 time point) gradually converges (0:30) and produces two distinct dots at the tips of small filopodia (1:00). Another filopodium is seen in-between with barely detectable GFP signal. The right filopodium moves laterally (1:00 through 2:00), and all three filopodia fuse (2:30), producing a single filopodium that protrudes extensively and acquires a club-like shape. Arrow points to the formation of a dorsal protrusion from a lateral filopodium. Linear fluorescence at the lower right side of the lamellipodium (0:30) gradually produces two filopodia by the 2:00 time point, which fuse (3:00), and the resulting structure translocates to the dorsal surface of lamella. (6.9 MB PDF) [file pbio.0050317.sg005.pdf]

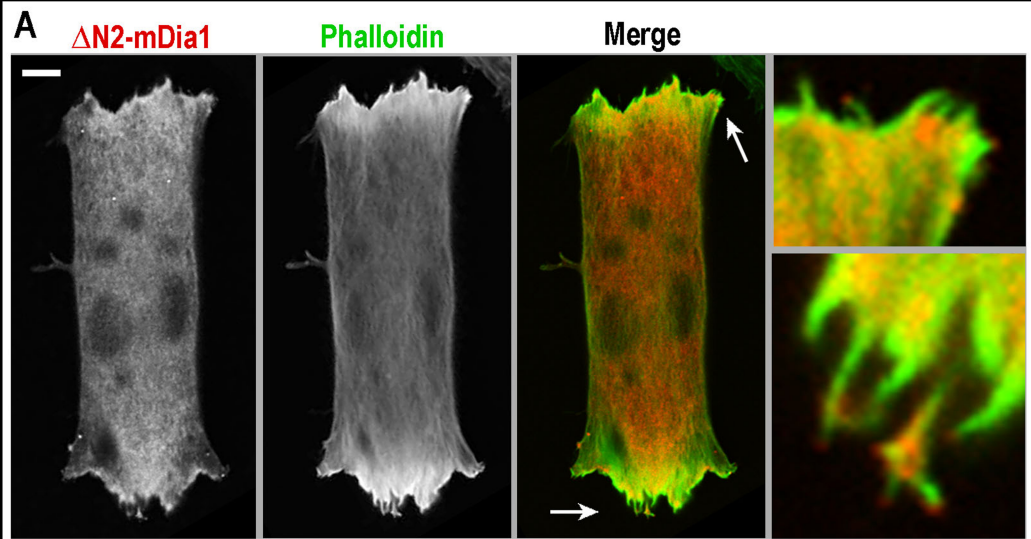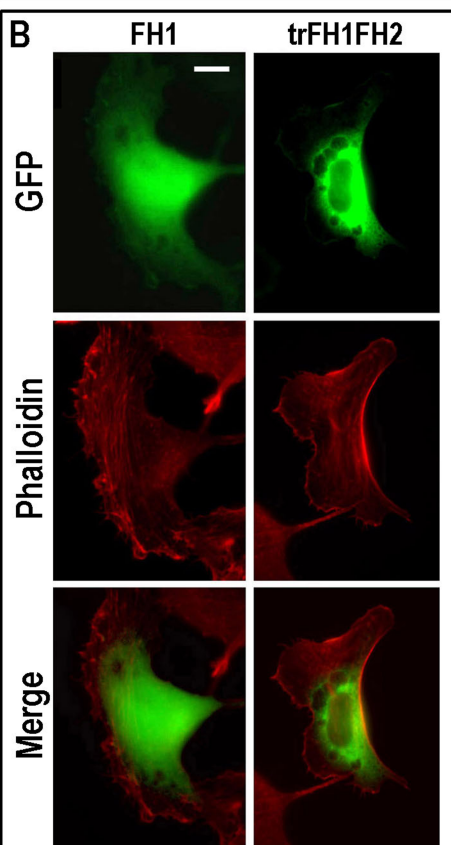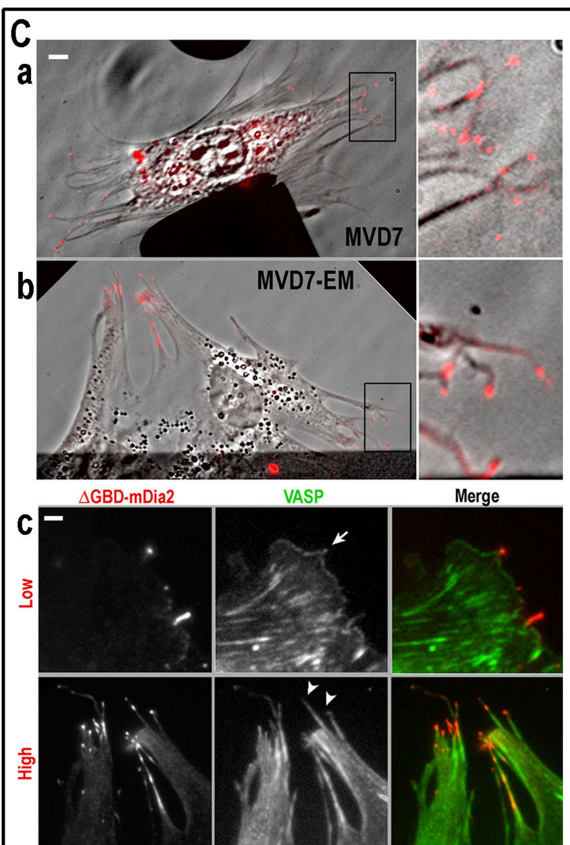

Supplement: Figure S6 — (A) Phenotype induced by expression of GFP-ΔN2-mDia1. GFP fluorescence of GFP-ΔN2-mDia1 (left) and F-actin enrichment (middle) are found throughout the cytoplasm of a bipolar cell as previously described in other cells [43]. Only very short, finger-like protrusions with slight enrichment of ΔN2-mDia1 at the tips could be observed at the cell edges abutted by actin bundles (right). No filopodial-like protrusions reaching significant length were observed. Arrow in the merged panel points to a region enlarged at right. (B) Expression of GFP-tagged mDia2 constructs in B16F1 cells. FH1 domain, residues 519–600 (FH1), and truncated FH1FH2, residues 519–909 (trFH1FH2) have cytoplasmic distribution and do not induce filopodia. Scale bars indicate 10 μm. (C) Filopodia induction does not depend on Ena/VASP proteins. Expression of GFP-ΔGBD-mDia2 (a and b) or mRFP1-ΔGBD-mDia2 (c) in Ena/VASP-deficient MVD7 cells (a) or MVD7 cells stably re-expressing GFP-Mena (MVD7-EM) (b) or transiently re-expressing GFP-VASP (c). (a and b) ΔGBD-mDia2 localizes to the membrane and induces filopodia equally well in MVD7 and MVD7-EM cells. (c) In cells expressing relatively low levels of ΔGBD-mDia2 (top row), re-expressed VASP is still occasionally present at filopodial tips (arrow), but is displaced to more proximal regions of filopodia (arrowheads) in highly expressing cells (bottom row). Scale bars indicate 5 μm in (a and b) and 10 μm in (c). (4 MB PDF) [file pbio.0050317.sg006.pdf]

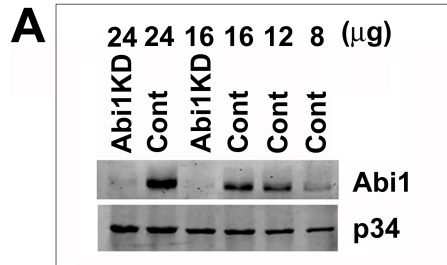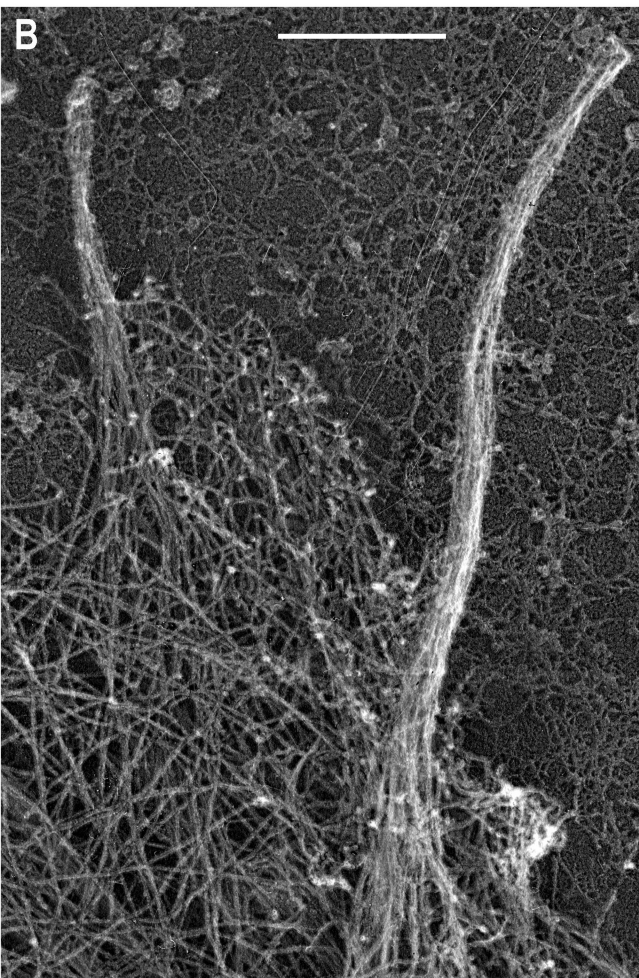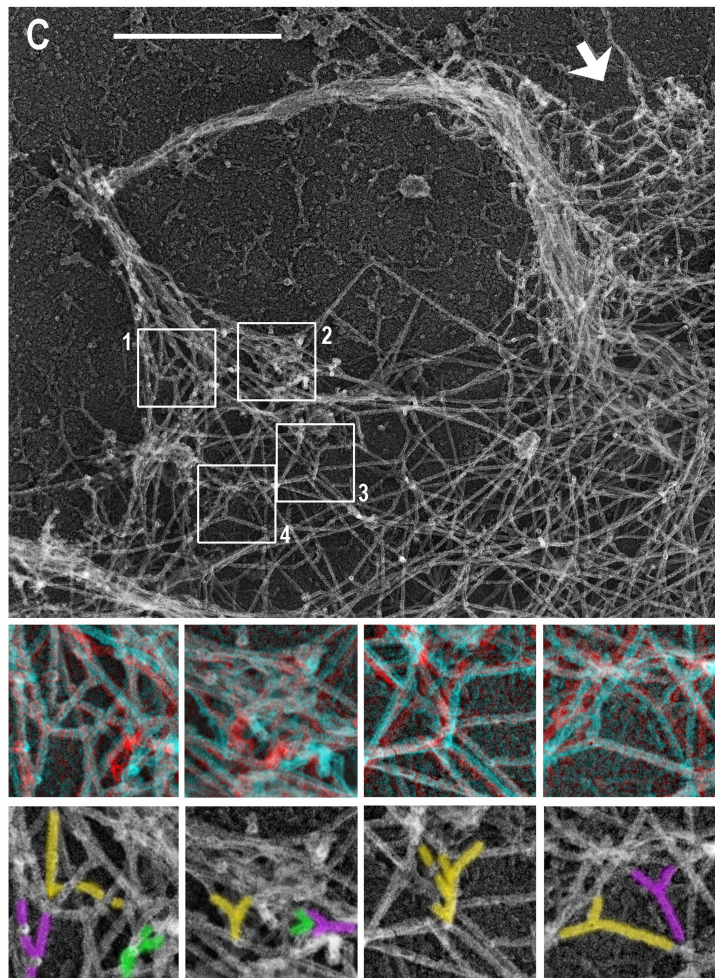

Supplement: Figure S7 — (A) Western blotting of lysates from control or Abi1KD HeLa cells. Amount of protein loaded is shown in μg. Expression of Abi1 is decreased by approximately 90% in Abi1KD cells, but Arp2/3 subunit p34-Arc (p34) is not changed. (B) EM of a peripheral region of a control HeLa cell. (C) EM of a peripheral region of Abi1KD cell with two filopodia. Boxed regions showing branched filaments in filopodial roots are enlarged at the bottom as 3D anaglyph images (right eye red) (top row), and as 2D images with branched filaments highlighted in color (bottom row). Although lamellipodia are grossly inhibited in these cells, small regions of dendritic network can be occasionally detected at cell edges (arrow). Bars indicate 0.5 μm. (8.8 MB PDF) [file pbio.0050317.sg007.pdf]
